# A guide to selecting psychological interventions that can be delivered by people who are not mental health specialists in low-resource settings

**DOI:** 10.1371/journal.pgph.0005123

**Published:** 2025-11-21

**Authors:** Ruta Rangel, Adam D. Brown, Jerome T. Galea, Sauharda Rai, Nicole Ross, Mansurat Raji, Pragya Shrestha, Bryan Cheng, Josephine Akellot, Brandon A. Kohrt

**Affiliations:** 1 Center for Global Mental Health Equity, The George Washington University, Washington, DC, United States of America; 2 Department of Psychology, The New School for Social Research, New York, New York, United States of America; 3 Department of Psychiatry, New York University School of Medicine, New York, New York, United States of America; 4 School of Social Work, University of South Florida, Tampa, Florida, United States of America; 5 Department of Mental Health, Bloomberg School of Public Health, Johns Hopkins University, Baltimore, Maryland, United States of America; 6 School Counseling and Psychological Wellbeing Unit, School of Education, Kathmandu University, Dhulikhel, Nepal; 7 Global Mental Health Lab, Columbia University, New York, New York, United States of America; 8 Mental Health and Psychosocial Support Department Digestive and Liver Disease Care Center, Mukono, Uganda; 9 Pukka Psychometric and Psychological Services, Kampala, Uganda; 10 Athena Institute, Vrije University Amsterdam, Amsterdam, the Netherlands; London School of Hygiene and Tropical Medicine Faculty of Epidemiology and Population Health, UNITED KINGDOM OF GREAT BRITAIN AND NORTHERN IRELAND

## Abstract

Globally, most individuals with mental health conditions lack access to specialized care. One strategy to bridge this gap is training people who are not mental health professionals (non-specialists) to deliver brief, manualized psychological interventions, which have demonstrated effectiveness across diverse settings. However, selecting the most suitable intervention for specific populations, contexts, and psychological needs in low-resource settings can be challenging due to the growing number of possible interventions and the differences in evidence of benefits across them. To facilitate this decision-making process, we provide an overview of ten psychological interventions that have demonstrated effectiveness in randomized controlled trials when delivered by non-specialists in low-resource settings for adults: Cognitive Processing Therapy, Common Elements Treatment Approach, Counseling for Alcohol Problems, Friendship Bench, Group Interpersonal Therapy, Healthy Activity Program, Problem Management Plus, Self-Help Plus, Step-by-Step, and the Thinking Healthy Programme. The interventions vary in settings where they have demonstrated effectiveness and the evidence for which conditions show benefit (e.g., depression, posttraumatic stress, substance use, general psychological distress). Considerations when selecting interventions also include the duration of treatment, ranging from 4 to 16 sessions, and the duration of training required for non-specialists, spanning from 5 to 12 days. This article, along with its visual summaries, serves as a guide to support selection of psychological interventions based on population needs, mental health conditions, and available resources.

## Introduction

Mental health conditions have consistently ranked among the top ten leading causes of disease burden worldwide for the past 30 years [1]. They account for an estimated 418 million disability-adjusted life years, highlighting their substantial impact on global health [2]. Unfortunately, of one billion people living with a mental health condition, few receive minimally adequate care [3]. The treatment gap is particularly stark in low- and middle-income countries (LMICs), where only one in 27 individuals with major depressive disorder (MDD) receives treatment, compared to one in five in high-income countries (HICs) [4].

The shortage of trained mental health professionals is a dominant barrier to accessible services. Approximately half of the global population resides in areas where fewer than one psychiatrist serves 200,000 people [5]. To address this treatment gap, the World Health Organization (WHO), the United Nations Children’s Fund (UNICEF), and other organizations advocate for task-sharing, which redistributes mental health care responsibilities from highly trained specialists to non-specialist providers [6–8]. Specialists, as well as non-specialist providers—such as community health workers, teachers, and nurses—can be trained to deliver evidence-based psychological interventions effectively [3,9,10]. Additionally, integrating mental health support into existing services, such as primary care and HIV programs, has been shown to be effective and cost-efficient, and is recommended over standalone mental health services [11]. Task-sharing approaches can also be incorporated into stepped-care models, which combine low-intensity treatments with higher-intensity interventions for those with greater mental health needs [12]. In these models, non-specialists provide initial psychosocial support, with referrals to mental health specialists when necessary [3].

In response to the increasing need for guidance on selecting and implementing psychological interventions, the Inter-Agency Standing Committee (IASC) has endorsed UNICEF’s Mental Health and Psychosocial Support Minimum Service Package (MHPSS-MSP) and WHO has published an implementation manual to support organizations in integrating evidence-based psychological interventions into their services [8,13]. WHO’s implementation manual outlines key steps, including planning, cultural adaptation, workforce training, identifying beneficiaries, and monitoring and evaluation [13]. The WHO implementation manual and MHPSS-MSP emphasize multi-sectoral collaboration and community engagement as essential components of successful implementation [8,13]. These documents provide a starting point for the implementation of manualized interventions in low-resource settings.

Although resources and guides have been created to guide the implementation of scalable mental health interventions, there has yet to be a systematic review aimed at identifying the most appropriate intervention based on *the context and population*. Therefore, this paper aims to complement the available guidance by providing additional insights to help organizations select and implement the most appropriate psychological interventions for their specific contexts. By synthesizing evidence from diverse sources, it is a practical resource for policymakers, program implementers, and researchers striving to close the global mental health treatment gap. Although there is an increasing number of meta-analyses which provide an estimation of benefit across existing studies [14,15], the goal here is to describe what works where and for whom to guide local selection of interventions.

For each intervention, we describe the mental health conditions it targets, the populations and settings where it has been implemented, who has been trained to deliver it, and the time required for training and intervention delivery. To support implementers in selecting the most appropriate intervention for specific target populations, we present visual infographics summarizing key features of each intervention.

## Methods

### Ethics statement

Ethics review was not required for this article, as it does not involve human subjects or original data collection. The article provides an overview of published effectiveness findings from randomized controlled trials (RCTs).

The selection of interventions was based on three primary criteria. First, each intervention must have demonstrated effectiveness in at least one RCT conducted in an LMIC. However, RCTs from HICs were included if they targeted refugee or asylum-seeking populations, as these settings often face similar resource constraints. Second, the interventions selected were designed for delivery by individuals without a formal background in mental health, such as lay persons, community health workers, teachers, and nurses. Third, this review focuses exclusively on interventions for adults; interventions designed specifically for children and adolescents are not included. Of note, there is growing literature regarding the implementation of these interventions outside the context of RCTs. However, the current overview is limited to the characteristics and findings in RCTs to focus on the implementation conditions under which the evidence for these interventions was generated.

Based on these criteria, we reviewed ten psychological interventions: Cognitive Processing Therapy (CPT), Common Elements Treatment Approach (CETA), Counseling for Alcohol Problems (CAP), Friendship Bench (FB), Group Interpersonal Therapy (IPT-G), Healthy Activity Program (HAP), Problem Management Plus (PM+), Self-Help Plus (SH+), Step-by-Step (SbS), and the Thinking Healthy Programme (THP). In addition, we provide an overview of Psychological First Aid (PFA); although it is not a multi-session intervention, it is widely used alongside the interventions described here.

To ensure applicability to real-world implementation, five subject matter experts in global mental health who implemented these interventions in Colombia, Nepal, Peru, and Uganda were consulted during the selection process. These experts, co-authors of this paper, contributed to interpreting findings and developing key considerations for selecting and implementing mental health interventions, which are presented in the discussion section.

### Psychological intervention domains

#### Psychological intervention rationale and content.

This section outlines the underlying rationale and key components of each psychological intervention. It describes the theoretical framework that informs the intervention, including the core principles guiding its development.

#### Intervention delivery and duration.

This domain describes the structure and timeline of each intervention, including the number of sessions, total duration, and key delivery characteristics. It also highlights variations in implementation, such as whether the intervention is conducted in person or remotely, and any other factors that influence accessibility and feasibility in different settings.

#### Helpers’ characteristics and training.

This section focuses on the individuals trained to deliver the interventions, referred to as “helpers.” Helpers may include peers, nurses, community health workers, primary care personnel, teachers, and other non-specialist providers. Additionally, this section examines the training process, specifying the duration required to equip non-mental health professionals with the skills necessary to deliver the intervention effectively and achieve meaningful mental health improvements. When available, information on monitoring fidelity is also provided.

#### Mental health conditions and populations served.

This domain provides an overview of the mental health conditions assessed in RCTs, such as depression and anxiety, and the conditions that showed improvement following intervention. All indications of improvement in mental health conditions refer to significant improvements. It also highlights any specific characteristics of the target population, such as individuals living with HIV/AIDS or refugees, to contextualize the applicability of each intervention.

### Psychological interventions

Table 1 below encompasses the ten psychological interventions. They are presented in order of the length of delivery, ranging from CAP (approximately 4 sessions) to THP (16 sessions).

**Table 1:** Characteristics of psychological interventions implemented in randomized controlled trials for adults in low- and middle-income countries or low-resource setting in high-income countries.

Intervention	Country: RCT	Helpers ^A^	Beneficiary Population	Inclusion Criteria	Sessions Completed ^B^	Final Assessment Period ^C^	Significant Mental Health Outcomes at Final Assessment	Non-Significant Mental Health Outcomes at Final Assessment
Counseling for Alcohol Problems	India: Nadkarni 2016, 2017 [28,30]	Community counsellors	Primary care patients	AUDIT ≥ 12	Mean = 2.8	12 months	Remission, abstinence in the past 14 days, recovery, percent of days being abstinent	Depression, suicidality, heavy drinking, functioning, perpetration of IPV
	Nepal: Jordans 2019 [31]	Community counsellors	Primary care patients	mhGAP AUD diagnosis by primary care worker	Mean = 3.1	12 months	No significant benefits	Harmful alcohol use, functional impairment
Self-Help Plus	Uganda: Tol 2020 [33]	Non-specialist facilitators	Refugees	K-6 ≥ 5	Uganda: 80% attended all 5 sessions	3 months	Depression, PTSD, psychological distress, functional impairment and subjective well-being, explosive anger	Psychological flexibility, personally identified problems
	Austria, Finland, Germany, Italy, UK: Purgato 2021 [36] & Turrini 2022 [37]	Non-specialist peers	Refugees and asylum seekers	GHQ-12 ≥ 3 and no diagnosis on MINI	Not reported	12 months	Depression, psychological distress, well-being	Prevention of mental health conditions
	Turkey: Acarturk 2022 [34]	Peer facilitators	Refugees	GHQ-12 ≥ 3 and no diagnosis on MINI	Not reported	6 months	Prevention of mental health conditions, depression, personally identified problems, quality of life	Psychological distress, PTSD, functional impairment, wellbeing
	China: Li 2024 [35]	Self-delivered	Healthcare workers	PSS-10 ≥ 15	Not reported	3 months	Depression, psychological stress, symptoms of insomnia, positive affect, and self-kindness	Anxiety, burnout
Problem Management Plus (individual)	Pakistan: Rahman 2016 [42]	Community health workers	Primary care patients	GHQ-12 ≥ 3 and WHODAS 2.0 ≥ 17	Mean = 4.2	3 months	Depression, anxiety, PTSD, personally identified problems, functional impairment	None
	Pakistan: Hamdani 2021 [41]	Master’s degree psychologists	Mental healthcare facility patients	GHQ-12 ≥ 3 and WHODAS 2.0 ≥ 17	Mean = 2.9	20 weeks	Depression, anxiety, PTSD, functional impairment	Personally identified problems, perceived social support
	Kenya: Bryant 2017 [43]	Community workers	Women affected by gender-based violence	GHQ-12 ≥ 3 and WHODAS 2.0 ≥ 17 and history of gender-based violence	Not reported	3 months	Psychological distress, personally identified problems, functional impairment	PTSD
	China: Zhang 2020 [44]	Nurses	Cancer patients	No mental health selection criteria	Not reported	3 months	Depression, anxiety, personally identified problems, functional impairment (all domains except for the cognition)	Other functional impairment domains (mobility, self-care, getting along, life activities, participation)
	Austria: Knefel 2022 [45]	Psychologists	Asylum seekers and refugees	RHS-15 ≥ 12 or RHS-15 distress scale ≥ 5	69% completed 5 sessions	7 weeks	Somatic symptoms, anxiety, insomnia, social dysfunction, depression, PTSD, quality of life, distress, personally identified problems	None
	Colombia: Perera 2022 [46]	Red Cross volunteers and supervisors	Migrants and refugees	WHO-5 = 29–74%	Not reported	Post-intervention	Quality of life, subjective wellbeing	Personally identified problems
	Netherlands: De Graaf 2023 [47]	Refugee peers	Refugees	K10 ≥ 16 and WHODAS 2.0 ≥ 17	84.5% participants attended at least 4 sessions	3 months	Depression, anxiety, PTSD	Personally identified problems, functional impairment
	Philippines: Luzano 2023 [48]	Facilitators	Students exposed to armed conflicts	GHQ-12 ≥ 2 and WHODAS 2.0 ≥ 17	Not reported	Post-intervention	Functional impairment anxiety, psychological distress, depression, PTSD, wellbeing	None
Problem Management Plus (group)	Pakistan: Rahman 2019 [49]	Community health workers	Women exposed to conflict	GHQ-12 ≥ 3 and WHODAS 2.0 ≥ 17	Mean = 3.8	3 months	Depression, anxiety, functional impairment, personally identified problems	PTSD, social support
	Nepal: Jordans 2021 [50]	Community members	Individuals affected by humanitarian disasters	WHODAS 2.0 ≥ 17 and presence of ‘heart-mind problems’ (local idiom of distress)	Nepal: 78% participants attended at least 4 sessions	3 months	Depression, psychological distress, local idiom of distress	Functional impairment, PTSD, social support, somatic symptoms
	Jordan: Bryant 2022 [51,52]	Community members	Refugees	K-10 ≥ 16 and WHODAS 2.0 ≥ 17	Mean = 4.0	12 months	None	Depression, anxiety, PTSD, functional impairment parenting behavior, child mental health, grief, prodromal psychotic symptoms, personally-identified problems
	Malawi: McBain 2024 [53]	Counsellors/ clinical officers	Patients of integrated chronic care clinics	PHQ-9 ≥ 10 and a clinical interview confirming depression with DSM-4 criteria	Median = 4.0	12 months	Depression, functional impairment	None
Step-by-Step	Lebanon: Cuijpers (2022) [57]	Community members	Refugees	PHQ-9 ≥ 10 and WHODAS 2.0 ≥ 17	32.2% finished all 5 sessions	3 months	Depression, anxiety, PTSD, subjective well-being, personally identified problems, functional impairment	None
	Lebanon: Cuijpers (2022) [58]	Community members	Lebanon residents	PHQ-9 ≥ 11 and WHODAS 2.0 ≥ 17	Mean = 1.7	3 months	Depression, anxiety, subjective well-being, personally identified problems, PTSD	Functional impairment
	China: Li 2024 [59]	Non-specialists	University students	PHQ-9 ≥ 5	Mean = 1.9	3 months	Subjective well-being, personally identified problems	Depression, anxiety
	Egypt: Buchert 2024 [60]	Non-specialists	Refugees	K-10 ≥ 16 and WHODAS 2.0 ≥ 17	36.8% completed 4 out of 5 sessions	3 months	Psychological distress, functional impairment	PTSD, personally identified problems
Friendship Bench	Zimbabwe: Chibanda 2016 [66]	Community health workers	Primary care patients	SSQ-14 ≥ 9	Median = 5.0	6 months	Local symptoms of distress, depression, anxiety, functional impairment, health related quality of life	None
	Zimbabwe: Haas 2023 [67]	Lay health workers	People with HIV^1^	SSQ-14 ≥ 9	88.1% attended all 6 sessions	12 months	None	ARV adherence, viral suppression, depression, local symptoms of distress
Healthy Activity Program	India: Patel 2016 & Woebong 2017 [69,70]	Community counsellors	Primary care patients	PHQ-9 ≥ 15	Median = 6	12 months	Depression	Suicidal behavior, disability, IPV, functional impairment
	Nepal: Jordans 2019 [31]	Community counsellors	Primary care patients	mhGAP depression diagnosis by primary care worker	Mean = 3.8	12 months	Depression, functional impairment	None
Group Interpersonal Therapy	Uganda: Bolton 2003 [75], Bass 2006 [74]	Community members	Rural population	Clinical interview with depression diagnosis according to DSM-4	Not reported	6 months	Depression, functional impairment	None
	Kenya: Meffert 2021 [76]	Nonspecialists	Women living with HIV^1^	MINI 5.0 with depression and PTSD diagnosis	Not reported	6 months	Depression, PTSD, functional impairment, intimate partner violence	None
	Turkey: Oral 2024 [77]	Clinical social workers	Nursing care facility workers	MBI emotional exhaustion ≥ 27 and depersonalization ≥ 10	Not reported	Post-intervention	Burnout, emotional exhaustion, depersonalization	Personal accomplishment
Common Elements Treatment Approach	Thailand: Bolton 2014 [80]	Lay counsellors (peers)	Refugees	Self-reported symptoms with adapted DSM algorithms for depression and PTSD using HSCL and HTQ	Mean = 9.7	Post-intervention	Depression, anxiety, PTSD, functional impairment, aggression	None
	Iraq: Weiss 2015 [81]	Community mental health workers	Survivors of systematic violence	Modified HTQ ≥ 36	Mean = 9.9	4 months	Depression, anxiety, PTSD, functional impairment	None
	Colombia: Bonilla-Escobar 2018 [82]	Lay psychosocial community workers	Survivors of systematic violence	TMHS ≥ 0.77 and gender-specific functional impairment	Quibdo´ mean = 5.9 Buenaventura mean = 7.2	Post-intervention	Buenaventura: Depression, anxiety, functional impairment Both municipalities: PTSD	Quibdo: Depression, anxiety, functional impairment
	Zambia: Murray 2020 [83]	Lay counsellors	Dyads of men engaged in hazardous alcohol use and women who experienced IPV	Dyads: females with SVAWS ≥ 38 and male partners with AUDIT ≥ 8	Not reported	12 months	Hazardous alcohol use, IPV	None
Cognitive Processing Therapy	Democratic Republic of Congo: Bass 2013 [89]	Psychosocial assistants	Survivors of sexual violence	Modified HSCL and PTSD Checklist ≥ 55 with functional impairment and interpersonal violence exposure	Mean = 8.5	6 months	Combined depression and anxiety, PTSD, functional impairment	None
	Iraq: Weiss 2015 [81]	Community mental health workers (medics and nurses)	Survivors of systematic violence	Modified HTQ ≥ 36	Not reported	4 months	Depression, PTSD	Functional impairment, anxiety
	Iraq: Bolton 2014 [88]	Community mental health workers	Survivors of systematic violence	Modified HSCL-25 ≥ 20	Not reported	5.5 months	Depression, functional impairment, PTSD, grief, anxiety	None
Thinking Healthy Program	Pakistan: Rahman 2008 [92]	Lady health workers	Mothers	Clinical interview with depression diagnosis according to DSM	Not reported	12 months	Depression, functional impairment, social support	None
	Pakistan: Sikander 2019 [93]	Mothers (peers)	Pregnant women	PHQ-9 ≥ 10	10.9	6 months	Social support	Depression, functional impairment
	India: Fuhr 2019 [94]	Mothers (peers)	Pregnant women	PHQ-9 ≥ 10	9.8	6 months	Depression, social support	Functional impairment

^A^Terminology used to describe ‘Helpers’ is based on the language used in the trial publications.

^B^Average number of sessions completed by participants is presented as reported in the trial, e.g., mean number sessions, percent completed a certain number of sessions.

^C^Final assessment period refers the number of months after the intervention. The latest assessment available is used, which may have been presented in a subsequent follow-up publication.

Notes: ACT – Acceptance and Commitment Therapy, AUDIT – Alcohol Use Disorders Identification Test, DSM – Diagnostic and Statistical Manual of Mental Disorders, GHQ-12 – The General Health Questionnaire, HSCL – Hopkins Symptom Checklist, HTQ – Harvard Trauma Questionnaire, IPV – Intimate Partner Violence, K6 – Kessler Psychological Distress Scale, K10 – Kessler Psychological Distress Scale, MBI – Maslach Burnout Inventory, mhGAP AUD – Mental Health Action Programme Alcohol Use Disorders, MINI – Mini International Neuropsychiatric Interview, PSS – Perceived Stress Scale, PTSD – posttraumatic stress disorder, RHS – Refugee Health Screener, SSQ – Shona Symptom Questionnaire, SVAWS – Severity of Violence Against Women Scale, TMHS – Total Mental Health Symptoms Scale, WHO-5 – World Health Organization Five Well-Being Index, WHODAS – World Health Organization Disability Assessment Schedule.

## Results

### Psychological first aid

#### Rationale and content.

Psychological First Aid (PFA) is not a multi-session psychological intervention; however, it is widely used as an immediate response to crises. We present it here because it is often used alongside manualized interventions for individuals requiring further support. PFA provides psychosocial assistance following disasters or large-scale crises, and is designed to support individuals in distress and facilitate connections to appropriate services [16]. Unlike targeted interventions for specific mental health conditions, PFA does not aim for long-term symptom reduction but instead promotes assessment of immediate needs, general well-being, and supports engagement with further care [16,17].

PFA is based on five core principles: ensuring safety, promoting calm, fostering connectedness, building self-efficacy, and promoting hope [18]. Different models instruct helpers assess safety, provide emotional support, and connect individuals to services. The WHO model follows a “look, listen, link” methodology: helpers look for safety risks and distress, listen to needs and concerns while using active listening techniques, and link individuals to appropriate services and social support [17]. By receiving training in structured action principles, helpers gain confidence in providing support during crises while maintaining their own well-being [17]. PFA training emphasizes the importance of referring individuals with urgent mental health needs, such as those experiencing severe psychiatric symptoms, suicidal ideation, or acute substance withdrawal—to appropriate care services [17].

#### Delivery and duration.

**Fig 1:**
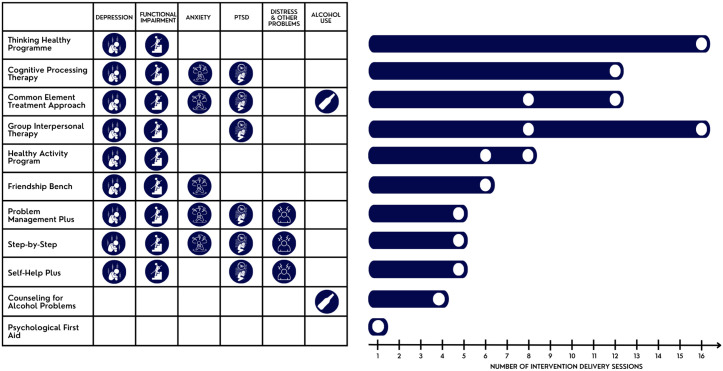
Length of interventions and conditions with demonstrated benefit. This list does not include all conditions that demonstrated the benefits listed in the table, only the conditions that improved based on the final assessment of the trial. The depression category includes depression and depressive symptoms; PTSD includes posttraumatic stress as well as post-traumatic stress disorder; problems refer to self-identified problems measured by PSYCHLOPS.

**Fig 2:**
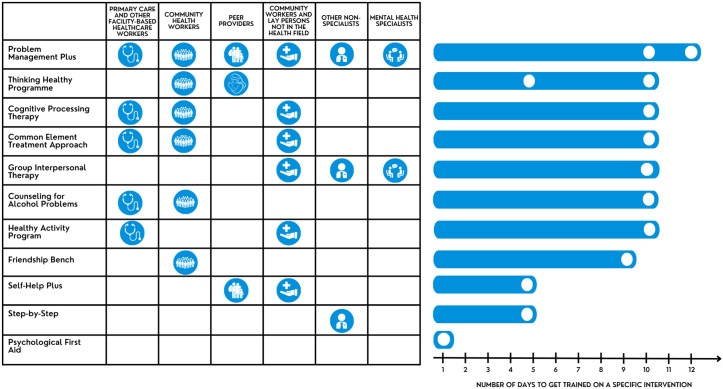
Summary of the training duration to become a helper for interventions.

There are numerous PFA curricula, including the WHO PFA, the National Child Traumatic Stress Network (NCTSN) PFA (used with adults and children/ adolescents), Johns Hopkins RAPID-PFA and International Federation of Red Cross and Red Crescent Societies (IFRC) [17,19–21]. PFA can be delivered on a brief ad hoc basis with variable duration based on the needs of the client [17]; one curriculum calls for a single 90 minute session, see Figs 1 and 2 [22].

#### Helpers’ characteristics and training.

Although PFA models share common strategies, they differ in their target audiences: WHO focuses on field workers in low-resource settings, NCTSN trains first responders, the Johns Hopkins model is designed for public health professionals, and IFRC focuses on their staff and volunteers [17,19–21]. Training duration varies significantly across models: the IFRC offers a self-paced 90-minute e-learning course [23], while the Johns Hopkins RAPID-PFA model includes a 6-hour training program that combines didactic presentations with practice sessions [20], a 3-hour version has also been used [24].

#### Mental health conditions and populations served.

Although PFA is widely implemented in low-resource settings, the ad hoc nature of PFA delivery makes it difficult to evaluate through RCTs, and it is therefore classified as an evidence-informed rather than evidence-based approach. PFA has been criticized for inconsistent delivery components (promotion of safety being used in studies more frequently than other PFA components), a high risk of bias, and inadequate follow-up (e.g., how long outcome improvements last), which have hampered establishing its evidence base [22,25,26]. The absence of rigorous effectiveness studies restricts the development of evidence-based implementation guidelines, ultimately affecting its scalability and sustainability [27]. Further research is needed to assess long-term feasibility, refine implementation strategies, and strengthen evidence-based guidelines for its implementation.

### Counseling for alcohol problems

#### Rationale and content.

**Fig 3:**
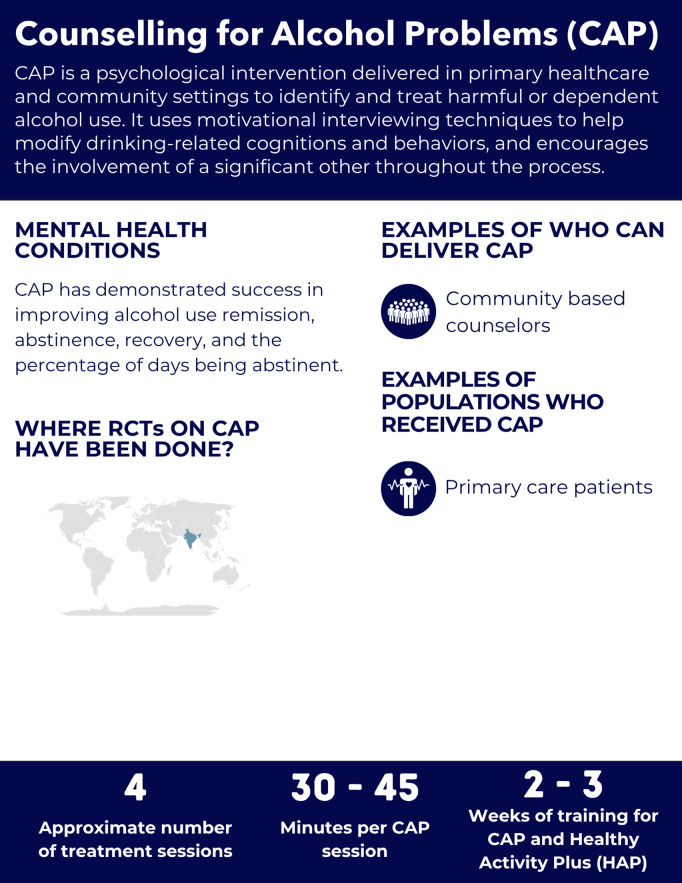
Overview of the Counselling for Alcohol Problems intervention.

Counseling for Alcohol Problems (CAP) is a psychological intervention used in primary healthcare settings to identify and treat harmful and dependent alcohol use (Fig 3) [28]. It employs motivational interviewing techniques to modify drinking-related cognitions and behaviors, encouraging the involvement of a significant other throughout the process [29]. CAP contains three phases: the initial phase, which involves assessing alcohol use and identifying drinking-related problems; the middle phase, focused on behavior change through skill development in alcohol refusal, handling peer pressure, problem-solving, and emotion regulation; and the final phase, which emphasizes relapse prevention and treatment completion [29]. RCTs evaluating CAP have been conducted in India [28,30] and Nepal [31].

#### Intervention delivery and duration.

The intervention is typically delivered in four 30–45 minute sessions, though duration may vary by client need [28,30]. During the initial session, the Alcohol Use Disorders Identification Test (AUDIT) is used to assess drinking habits, and the client receives psychoeducation on alcohol-related consequences [29]. If the client agrees to pursue behavioral change, an individualized action plan is developed [29]. The subsequent sessions involve monitoring progress, refining strategies, and implementing relapse prevention techniques [29].

#### Helpers’ characteristics and training.

CAP has been delivered by community-based counsellors working in a primary care setting [28,30,31]. The CAP manual does not specify educational requirements for helpers, but in India, the counsellors had no background in mental health, had at least a secondary school education and fluency in local languages; in Nepal, counsellors had at least a high school education [28,30,31].

Training durations varied across studies. In all studies, CAP training lasted two weeks or 10 days, but included training on both CAP and the Healthy Activity Program (HAP) [28,30,31]. Fidelity was assessed using several components, including intervention completion rates, CAP quality scores rated by peers, and supervisor ratings of 10% randomly selected pre-recorded sessions [28].

#### Mental health conditions and populations served.

The two RCTs on CAP were conducted among primary care patients [28,30,31]. In India, the inclusion criteria were harmful drinking, measured by AUDIT (score ≥ 12); in Nepal, the inclusion criteria were alcohol use disorder determined by a health worker based on the Mental Health Gap Action Program (mhGAP) guidelines [31]. Follow-up was at 3 and 12 months in India [28,30] and Nepal [31].

*Depression and Anxiety, PTSD, Distress, Functioning, Other Psychological Outcomes:* Not evaluated.

*Functioning:* In Nepal and India, the functional impairment did not significantly improve, assessed by the WHO Disability Assessment Schedule (WHODAS 2.0) [28,30,31].

*Substance Use Conditions:* In India, the study demonstrated significant improvements in remission rates at three months, assessed by the AUDIT < 8. Abstinence rates in the past 14 days were also higher in the intervention group at three months (41% vs. 18%). These improvements were sustained at the 12-month follow-up, where remission rates reached 54.3% with CAP, compared to 31.9% in the control group and abstinence rate was 45.1% in the intervention arm compared to 26.4% in the control [28,30]. CAP also demonstrated benefits in recovery and in the percent decrease of days being abstinent at 12 months follow-up [28,30]. CAP did not significantly impact alcohol consumption on drinking days, work-related disability, or intimate partner violence [28,30].

In Nepal, CAP was compared to a control group receiving mhGAP-based interventions delivered by primary health workers. Unlike the India study, CAP did not show improvement in the AUDIT scores [31].

### Self-help plus

#### Rationale and content.

**Fig 4:**
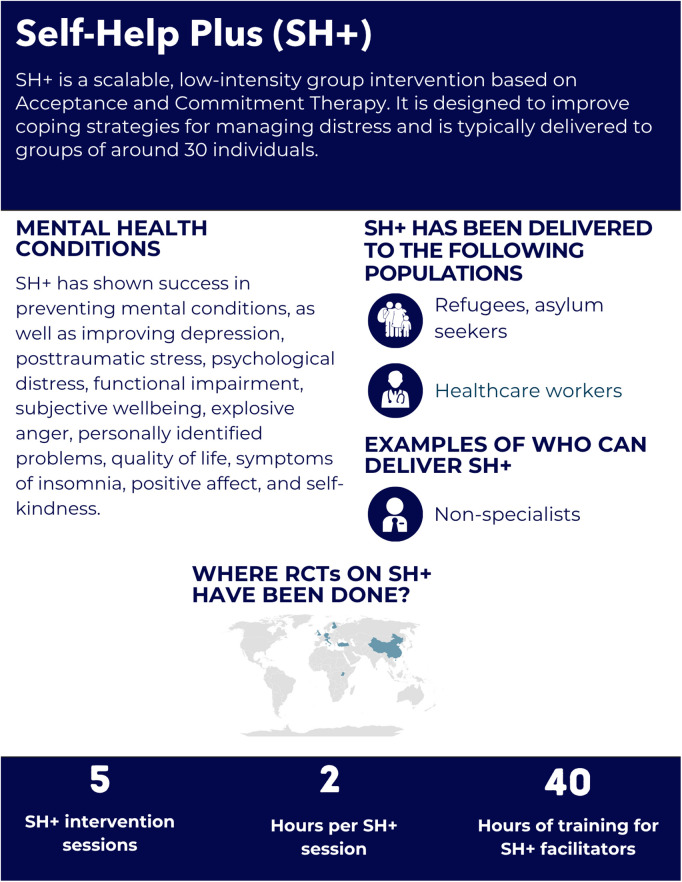
Overview of the Self Help Plus intervention.

Self-Help Plus (SH+) is a low-intensity stress management intervention based on Acceptance and Commitment Therapy, designed to be delivered to groups of approximately 30 individuals (Fig 4) [32]. RCTs on SH+ have been conducted in Uganda [33], Turkey [34], China [35], and two RCTs involved several countries: Turkey, Italy, Germany, Austria, Finland, and UK [36,37].

#### Intervention delivery and duration.

SH+ consists of five 2-hour sessions delivered in a workshop format where participants listen to pre-recorded audio material while facilitators guide discussions using a guide [32]. Each session includes approximately 50 minutes of audio and 30–40 minutes of interactive activities [32]. Participants receive an illustrated self-help book, “Doing What Matters in Times of Stress,” which reinforces key concepts [32]. The core SH+ strategies include grounding (staying present), unhooking (detaching from distressing thoughts), acting on values, being kind (to oneself and others), and making room (accepting difficult emotions) [32]. SH+ can be provided as a stand-alone intervention or with other tools as the first step in the stepped care model, where individuals start with low-intensity support and progress to more intensive interventions if needed [32].

#### Helpers’ characteristics and training.

In SH + , helpers are referred to as ‘facilitators,’ and are not required to have prior mental health experience [32]. The training is 40 hours focused on communication, confidentiality, and validation [32]. Facilitators are recommended to share a cultural and linguistic background with participants and have at least a high school education [32]. Weekly supervision is recommended after completing the training, though the frequency may vary based on facilitator experience and participant needs [32]. Some RCTs provided additional support; for example, in Western Europe, facilitators had access to clinical psychologists and SH+ expert trainers who provided supervision and ensured intervention fidelity [36,37]. In most of the reviewed studies, fidelity was assessed through supervisor-completed adherence checklists and observation (ranging between 10–20% of the sessions) [33,36,37], or through self-reported measures [34].

#### Mental health conditions and populations served.

The reviewed RCTs focused on delivering SH+ to South Sudanese refugees in settlements in Uganda [33], refugees and asylum seekers in Western Europe [36,37], healthcare workers through social media platforms in China [35], and Syrian refugees in Turkey [34]. In Uganda, the selection criteria were moderate psychological distress, measured by the Kessler 6 (K6 ≥ 5) [33]. In Western Europe and Turkey, the psychological distress, measured by the General Health Questionnaire (GHQ-12 ≥ 3), and no presence of mental disorder according to the Mini International Neuropsychiatric Interview (MINI) [34,36,37]; in China the selection criteria was high level of stress, assessed by the Perceived Stress Scale (PSS-10 ≥ 15) [35]. In Turkey and Western Europe, the studies assessed prevention effects at 6 and 12 months follow-up [34,36,37]; whereas studies in Uganda and China measured the treatment effects at 3 months, and 2 weeks, 1 and 3 months follow-up, respectively [33,35].

*Depression and Anxiety:* SH+ showed benefits in reducing depression symptoms in all studies. Depression symptoms significantly improved in Uganda [33], China [35], and Western Europe (after the intervention and at 12 months follow-up), assessed by the Patient Health Questionnaire (PHQ-9) [36,37]. In Turkey, SH+ demonstrated prevention effects of depression, anxiety, and other mental health conditions, assessed by MINI at 6 months follow-up compared to the control group [34]. In Western Europe, SH + did not demonstrate significant prevention effects at 6 or 12 months, as assessed by the MINI [36,37]. In China, SH+ impact on reducing anxiety was not significant, as measured by the Generalized Anxiety Disorder-7 (GAD-7 > 10) [35].

*PTSD:* SH+ showed benefit in reducing PTSD symptoms in 1 out of the 3 studies in which it was evaluated. In Uganda, PTSD symptoms improved three months post-intervention, as assessed by the PTSD Checklist-Civilian (PCL-6) [33]. In Western Europe and Turkey, no significant improvements were found in trauma symptoms at 12 and 6 months, respectively, measured by the PCL-5; however, SH+ demonstrated prevention effects for the diagnosis of PTSD [34,36,37].

*General Psychological Distress:* SH+ showed benefit in reducing general psychological distress in all studies in which it was evaluated. Psychological distress significantly improved in Turkey post-intervention but not at 6 months follow-up [34], Western Europe (post-intervention and at 12 months follow-up) [36,37], assessed by the GHQ‐12 ≥ 3 and Uganda [33], measured by the K6 ≥ 5. In China, psychological stress significantly improved, as measured by the PSS-10 ≥ 15 [35]. Personally identified problems, assessed by the Psychological Outcome Profiles (PSYCHLOPS), showed significant improvement in Turkey at 6 months follow-up [34], as well as in Western Europe [36] post-intervention, but not at the follow-up. No effects were found in Uganda [33].

*Substance Use Conditions:* Not evaluated.

*Functioning and Quality of Life*: Functional impairment and subjective wellbeing, assessed by the WHODAS 2.0 and WHO Wellbeing Index (WHO-5), respectively, improved in Uganda [33] and wellbeing in Western Europe at 6 and 12 months follow-up [36,37], but no improvements were found in Turkey at 6 months follow-up [34]. Additionally, quality of life and general health, assessed by the European Quality of Life 5‐Dimensions 3‐Level (EQ‐5D‐3L), showed improvements in Turkey at 6 months follow-up [34], but no effects were found in Western Europe [36,37].

*Other Outcomes:* Symptoms of insomnia, positive affect, and self-kindness, as measured by the Insomnia Severity Index, Positive and Negative Affect Scale, and Self-Compassion Scale, respectively, showed benefits in China [35]. Finally, reductions in explosive anger symptoms were observed; however, no significant effects on psychological flexibility were found in Uganda, measured by the Acceptance and Action Questionnaire (AAQ-II) [33].

### Problem management plus

#### Rationale and content.

**Fig 5:**
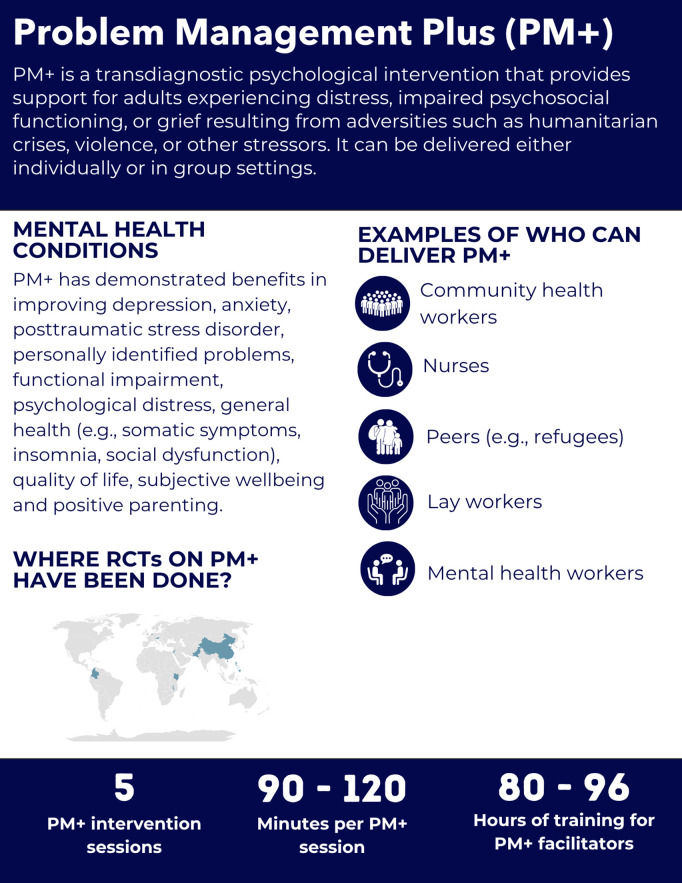
Overview of the Problem Management Plus intervention.

Problem Management Plus (PM+) is a transdiagnostic (anxiety and depression) psychological intervention that provides support for adults experiencing distress, impaired psychosocial functioning, and grief due to adversities such as humanitarian crises, violence, and other stressors (Fig 5) [38–40]. Individual PM + RCTs have been conducted in Pakistan [41,42], Kenya [43], China [44], Austria [45], Colombia [46], Netherlands [47], and the Philippines [48]. The Group PM + RCTs have been done in Pakistan [49], Nepal [50], Jordan [51,52], and Malawi [53].

#### Intervention delivery and duration.

PM + is delivered either through individual (five 90-minute sessions) or group (five 2-hour sessions) formats, following the WHO manuals [38,39]. Group sessions include up to 12 participants and two facilitators. When feasible, participants are encouraged to be of the same sex and share similar cultural, religious, or political backgrounds [39]. In addition, Individual PM + can be delivered in-person and remotely, offering flexibility across settings [54,55].

#### Helpers’ characteristics and training.

Three RCTs indicated that the recruited helpers did not have professional training or prior experience in mental health care [42,43,50]. Facilitators included lay workers in Pakistan, Kenya and Jordan [42,43,49,51,52], Red Cross volunteers and supervisors in Colombia, previously trained on PFA [46], facilitators in the Philippines [48], community workers Nepal [50], Syrian refugees (peers) in the Netherlands [47], nurses in China [44] and counsellors/ clinical officers in Malawi [53]. Two RCTs limited recruitment to helpers who had a background in mental health in Pakistan and Austria [41,45].

Training requires 80 hours (10 days) for individual PM+ and 96 hours (12 days) for group PM +  [38,39]. Trainees complete supervised practice with at least two clients before independent delivery [38]. Supervision continues post-training, with weekly sessions lasting 1–2 hours for individual facilitators and 2–3 hours for group facilitators [38,39].

Fidelity to Individual PM + was assessed through supervisor-completed checklists evaluating adherence to PM+ strategies in Pakistan and Kenya [42,43]; in Austria, fidelity relied on self-reports and supervision sessions [45]; whereas in the Netherlands, fidelity was evaluated using self-reported measures and supervisor reviews of pre-recorded session audios [47]. For Group PM + , fidelity was assessed through independent observers who assessed randomly selected sessions for intervention components; supervisors then reviewed these ratings to determine session quality in Pakistan [49]. In Nepal, supervisors completed checklists for two sessions per group to assess both fidelity and competency [50]. In Jordan and Malawi, fidelity was measured through supervisor assessments using checklists [51,53].

#### Mental health conditions and populations served.

Individual PM + was delivered to primary care clinic [42] and mental healthcare facility patients in Pakistan [41], women exposed to gender-based violence in Kenya [43], cancer patients in hospitals in China [44], Afghan refugees and asylum seekers in nongovernmental organizations in Austria [45], Venezuelan migrants and refugees and Colombian returnees in Red Cross offices or participant homes in Colombia [46], Syrian refugees in either digital or hybrid formats in the Netherlands [47], and students exposed to armed conflicts in the Philippines [48].

Group PM + has been delivered to conflict-affected women in Pakistan [49], individuals affected by humanitarian disasters (e.g., landslides and flooding) in Nepal [50], Syrian refugees in refugee camps in Jordan [51,52], and patients of integrated chronic care clinics for various conditions, such as hypertension, diabetes, etc. in Malawi [53].

The selection criteria for Individual PM+ were common mental health conditions (GHQ-12 ≥ 3) and functional impairment (WHODAS 2.0 ≥ 17) in Pakistan [42]; in another study in Pakistan the selection criteria were psychological distress (GHQ-12 > 2) and functional impairment (WHODAS 2.0 > 16) [41]; in Kenya, history of gender based violence, distress (GHQ-12 ≥ 3) and functional impairment (WHODAS ≥ 17) [43]; no mental health selection criteria were included in the China study [44]; in Austria, psychological distress, measured by Refugee Health Screener (RHS-15 ≥ 12 or the RHS-15 distress scale ≥ 5) [45]; in Colombia, subjective wellbeing (WHO-5, score, more than 28 but ≤ 74) [46], in the Netherlands, psychological distress, assessed by the Kessler Psychological Distress Scale (K10 > 15) and impaired functioning (WHODAS 2.0 > 16) [47]; in the Philippines, distress (GHQ-12 ≥ 2) and functional impairment (WHODAS 2.0 ≥ 17) [48].

In Pakistan the treatment was assessed at 3 months follow-up [42], and at 7 and 20 weeks after baseline follow-up [41], 3 months in Kenya [43], China [44], and the Netherlands [47].

Contextual adaptations exist; for instance, a sixth session was added in Austria to address anger management and post-migration difficulties; therefore, the post-treatment effects were assessed 7 weeks after the baseline; more on cultural adaptation can be found in the discussion section [45]. No follow-up assessment was included in the Colombia [46] or the Philippines studies [48].

For Group PM + , the selection criteria were common mental health conditions (GHQ-12 ≥ 3) and functional impairment (WHODAS 2.0 ≥ 17) in Pakistan [49]; psychological distress assessed using local idiom of distress (“heart–mind problems”) and functional impairment (WHODAS 2.0 > 16) in Nepal [50]; psychological distress (K-10 ≥ 16) and functional impairment (WHODAS ≥ 17) in Jordan [51,52]; recent diagnosis of depression (PHQ-9 ≥ 10) and depression criteria based on Diagnostic and Statistical Manual of Mental Disorders (DSM-4) in Malawi [53].

The Group PM + RCTs follow-up period was 3 months in Pakistan [49] and Nepal [50], 3 and 12 months in Jordan [51,52], and assessments were conducted at 12 months follow-up in Malawi [53].

*Depression and Anxiety:* Individual PM+ demonstrated benefit for depression in all studies in which it was evaluated. Depression improved in Pakistan as measured by the depression subscale of the Hospital Anxiety and Depression Scale (HADS) and PHQ [41,42]; China [44], measured by HADS, in the Netherlands [47], using the depression subscale of Hopkins Symptom Checklist-25 (HSCL-25), the Philippines [48], assessed with the PHQ-9 ≥ 10 and Austria [45], assessed by the General Health Questionnaire 28 (GHQ-28), which measures somatic symptoms, anxiety, depression, insomnia and social dysfunction. Anxiety symptoms improved in all studies in which it was evaluated, with benefits observed in the Netherlands [47], assessed by the anxiety subscale of the HSCL-25; in Pakistan [41,42] and China [44], measured by the anxiety subscale of HADS; Austria, evaluated by the GHQ-28 [45], and in the Philippines [48], measured by the GAD-7.

For Group PM + , depression symptoms significantly improved in Pakistan [49], assessed by the HADS and the PHQ-9 ≥ 10, and Jordan, measured by the HSCL-25 at 3 but not 12 months follow-up [51,52]. In Nepal [50] and Malawi, depression symptoms also improved, evaluated by the PHQ-9 [53]. Anxiety improved in Pakistan [49], measured by the HADS, but no improvements were found in Jordan [51,52].

*PTSD:* For Individual PM + , PTSD symptoms showed significant improvement in Pakistan [41,42], the Netherlands [47], the Philippines [48], as assessed by the PCL-5 and Life Events Checklist for DSM-5 (LEC-5), and in Austria [45], measured by the International Trauma Questionnaire (ITQ). No such benefits were observed in Kenya, assessed by PCL [43]. However, for Group PM + , none of the 3 studies in which PTSD was evaluated showed benefit in Jordan [51,52], Nepal [50], and Pakistan [49], as measured by PCL-5.

*General Psychological Distress:* For Individual PM + , distress and wellbeing improved in Kenya [43] and the Philippines [48], assessed by the GHQ-12, and in Austria [45], as assessed by the Post-Migration Living Difficulties (PMLD) Checklist. Subjective wellbeing significantly improved in Colombia [46], as measured by the WHO-5. Individual PM+ also led to improvements in personally identified problems in Pakistan [42], Kenya [43], Austria [45], and China [44]; however, no such effects were found in Pakistan [41], Colombia [46] and the Netherlands [47].

For Group PM + , generalized psychological distress improvement was observed in Nepal [50], as measured by the GHQ-12, together with the local idiom of distress (“heart–mind problems”). Group PM + has also significantly improved personally identified problems in Pakistan [49], and Jordan right after the intervention but not at 12 months follow-up [51], as measured by the PSYCHLOPS.

*Substance Use Conditions:* Not evaluated.

*Functionality and Quality of Life:* Individual PM+ showed benefits for functional impairment in Kenya [43], the Philippines [48], and Pakistan [41,42], measured by the WHODAS 2.0 ≥ 17 and WHO Disability Assessment Scale (WDS) [42]; however, no statistically significant benefits were found in the Netherlands [47]. In China, the mobility, self-care, getting along, life activities, and participation components of the WHODAS 2.0 showed significant improvement, except for the cognition domain [44]. Quality of life improved in Austria [45] and Colombia [46] based on the WHO Quality of Life questionnaire (WHOQOL-BREF). For Group PM + , functional impairment improvement was observed in Pakistan [49], and Malawi [53], as measured by the WHODAS 2.0 (score ≥ 17 in Pakistan); however, no benefits were found in Nepal [50] or Jordan [51,52].

*Other Outcomes:* Perceived social support, measured by the Multidimensional Scale of Perceived Social Support (MSPSS) did not improve in the intervention arm in Nepal [50] and Pakistan [41,49], as well as somatic symptom severity, measured by the Somatic Symptom Scale 8 in Nepal. Finally, grief, prodromal psychotic symptoms, parenting behavior, and children’s mental health, measured by Prolonged Grief Disorder (PG-13), Prodromal Questionnaire-16 (PQ-B), Alabama Parenting Questionnaire-42 (APQ), and the Pediatric Symptoms Checklist (PSC), respectively, in Jordan, showed no significant improvement [51].

### Step-by-step

#### Psychological intervention rationale and content.

**Fig 6:**
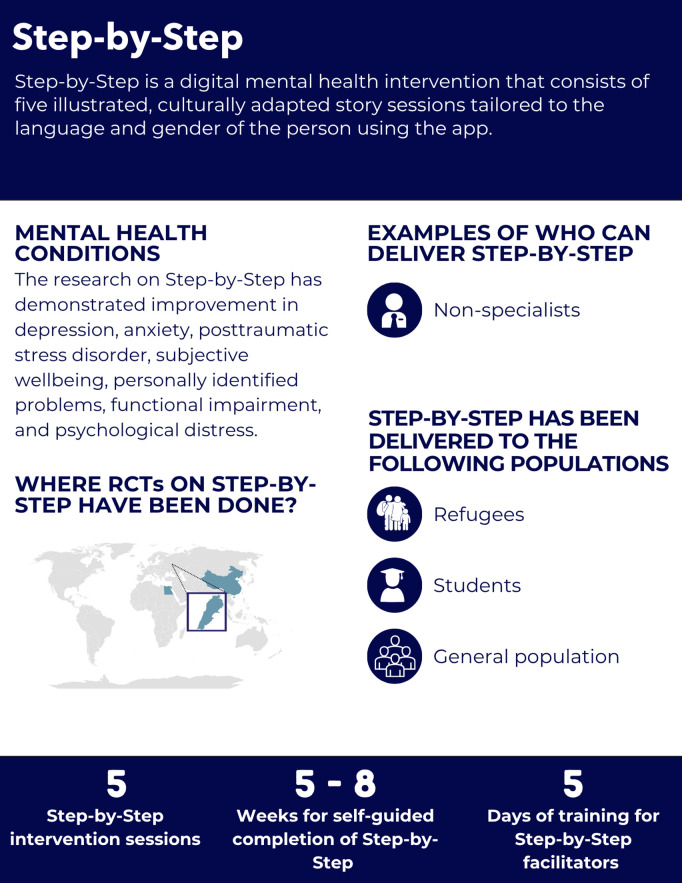
Overview of the Step by Step intervention.

Step-by-Step (SbS) is a digital mental health intervention developed by WHO in collaboration with Lebanon’s Ministry of Public Health and partners (Fig 6) [56]. SbS RCTs have been conducted in Lebanon [57,58], China [59], and Egypt [60].

#### Intervention delivery and duration.

The intervention consists of five illustrated, culturally adapted story sessions tailored to the language and gender of the person using the app [57–60]. Each session narrates a story of an individual seeking help for depression while teaching coping strategies [61]. The self-guided five digital sessions are completed over 5–8 weeks [57].

#### Helpers’ characteristics and training.

Helpers, or e-helpers, focused on supporting clients emotionally and providing technical assistance, received five days of active listening and problem-solving training under the supervision of mental health professionals [57,58]. Their role included risk assessment of self-harm, child abuse or gender based violence, providing appropriate referrals and brief weekly (15-minute) support via phone, messaging, or email [57,58,60]. In Lebanon and Egypt, they were individuals with a background in psychology but without prior experience in delivering mental health support [57–60].

Fidelity was monitored either through checklists used to rate 5% of all received calls and messages [57,58], or through weekly supervision sessions [59].

#### Mental health conditions and populations served.

The target population for this intervention included Syrian refugees in Lebanon [57] and Egypt [60], Lebanese residents [58] and university students in China [59]. Inclusion criteria on mental health instruments were moderate to severe depression, measured on the PHQ-9 ≥ 10 [57] and PHQ-9 > 10 [58], functional impairment (WHODAS > 16) in Lebanon [57,58], mild depression symptoms in China (PHQ-9 ≥ 5) [59], psychological distress, (K10 > 15) and functional impairment (WHODAS 2.0 > 16) in Egypt [60]. Outcomes were evaluated at 3 months follow-up in all studies [57–60].

*Depression and Anxiety:* Depression symptoms significantly improved in Lebanon (PHQ-9 ≥ 10) and the effects were sustained at 3 months follow-up [57,58]. In China, depression improved post-intervention; however, the effects diminished by 3 month follow-up [59]. Anxiety improved in Lebanon [57,58], assessed by the GAD-7 with sustained effects at 3 months follow-up, and in China post-intervention but not at follow-up [59].

*PTSD:* PTSD symptoms were significantly reduced in Lebanon [57,58]; however, no significant effects were found in Egypt [60], as assessed with the PCL-5.

*General Psychological distress*: Psychological distress, assessed by the HSCL-25, demonstrated a small improvement in psychological distress in Egypt and the effects were sustained at 3 months follow-up [60]. Subjective well-being improved in China [59] and Lebanon [57,58], measured by the WHO-5. Personally identified problems, as measured with the PSYCHLOPS, showed benefits in Lebanon [57,58]. In China, at 3 months follow-up, there was a significant improvement in personally identified problems on the per-protocol analysis but not according to the intention-to-treat analysis [59]. No such effects were observed in Egypt [60].

*Substance Use Conditions:* Not evaluated.

*Functionality:* Functional impairment improved in Egypt [60], and one study in Lebanon [57]; however, no sustained effects were found in the second study in Lebanon [58], as measured by the WHODAS > 16.

### Friendship Bench

#### Psychological intervention rationale and content.

**Fig 7:**
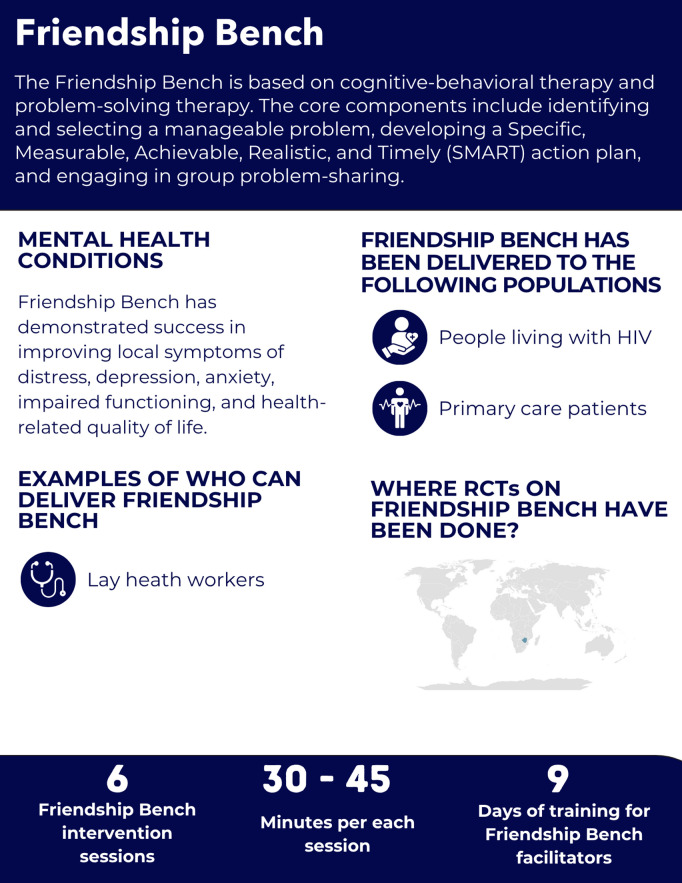
Overview of the Friendship Bench intervention.

The Friendship Bench was developed in Zimbabwe to address the high prevalence of common mental health conditions, such as depression and anxiety (Fig 7) [62]. Initially delivered outdoors on benches near primary health care clinics [63,64], The Friendship Bench (FB) is based on cognitive-behavioral therapy and problem-solving therapy [63]. The core components include Opening the Mind (*kuvhura pfungwa*)—identifying and selecting a manageable problem, Uplifting (*kusimudzira*)—developing a SMART (Specific, Measurable, Achievable, Realistic, and Timely) action plan, and Strengthening (*kusimbisa*)—engaging in group problem-sharing [65]. Rather than following a diagnosis-driven model, it empowers clients to manage their problems and strengthen coping skills by guiding them through a series of questions focused on identifying challenges and selecting practical solutions [66]. In addition to sessions delivered in primary care, home visits are recommended during later sessions, and clients are encouraged to join weekly *Kubatana Tose* circles for peer support [65]. The intervention fosters community support and belonging [66]. To date, two RCTs have been conducted in Zimbabwe [66,67].

#### Intervention delivery and duration.

The Friendship Bench consists of six 30–45-minute sessions [63]. Sessions 1–3 are dedicated to building rapport, identifying the problems and developing an action plan to address those challenges [65]. Sessions 4–6 focus on reviewing and adjusting the action plan [65]. The RCTs were evaluations of the Friendship Bench delivered in primary care and antiretroviral therapy (ART) clinics [66,67].

Fidelity was assessed using checklists for pre-recorded sessions and by tracking session attendance [66,67].

#### Helpers’ characteristics and training.

The cadre described in the two RCTs were lay health workers [66,67]. Training lasts 9 days or two weeks [66], covering key competencies such as confidentiality, empathy, active listening, non-judgmental engagement, and guiding clients through the process [65]. To ensure the quality of the delivered care and support for the helpers, weekly supervision sessions are recommended [65,68].

#### Mental health conditions and populations served.

FB targets common mental disorders (CMDs) that include anxiety and depressive conditions, locally termed “*kufungisisa*” (thinking too much), including depression and anxiety [65]. These conditions are often linked to psychosocial stressors or genetic predispositions and may co-occur with post-traumatic stress disorder, panic disorder, cognitive disorders, HIV, and substance use disorders [65]. In the reviewed RCTs, the intervention was delivered to primary care attendees [66] and individuals living with HIV in a rural setting [67]. Inclusion criteria were scoring ≥ 9 on the locally validated Shona Symptom Questionnaire (SSQ-14), used to assess CMD in both studies [66,67]. The follow-up time was 6 months [66] and 12 months [67].

*Depression and Anxiety:* Depression severity was measured both by the SSQ-14 and the PHQ-9 ≥ 11 and demonstrated improvements in Chibanda’s study [66], but no such effects were found in Hass’ study [67]. Anxiety, measured by the GAD-7 [66], improved in Chibanda’s study [66].

*PTSD, Substance Use Conditions:* Not evaluated.

*General Psychological Distress:* The intervention significantly reduced local symptoms of distress at 3 [67], 6 [66,67] and 9 months [67], but not at 12 months follow-up [67].

*Functioning:* FB demonstrated significant improvement in functional impairment, as assessed by the WHODAS 2.0 [66]. Health-related quality of life (EQ-5D) also significantly improved [66].

*Other Outcomes:* The intervention had no impact on antiretroviral medication adherence or viral suppression [67].

### Healthy activity program

#### Psychological intervention rationale and content.

**Fig 8:**
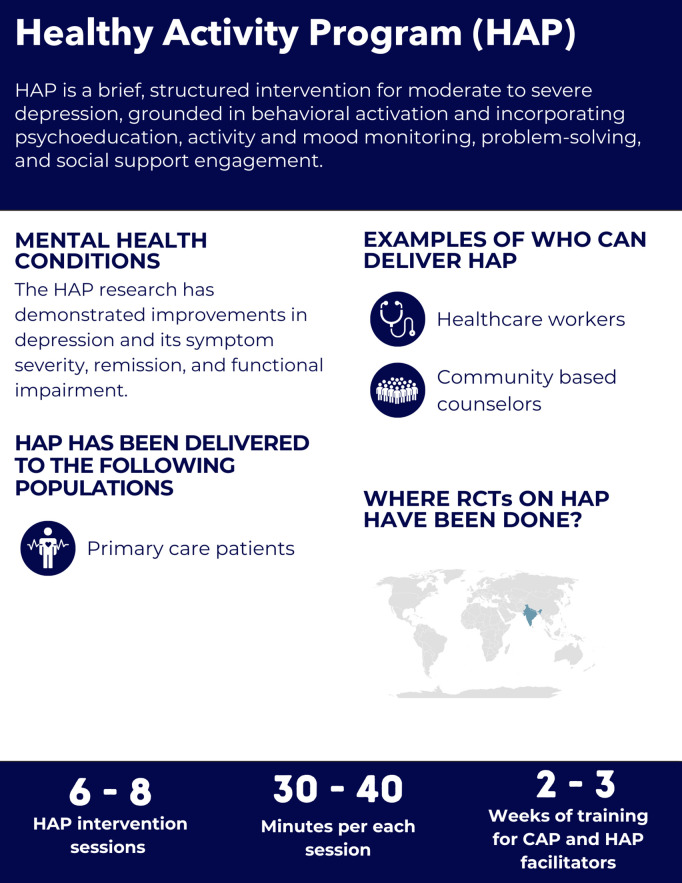
Overview of the Healthy Activity Program intervention.

The Healthy Activity Program (HAP) was developed under the Program for Effective Mental Health Interventions in Under-Resourced Health Systems (PREMIUM) to provide a culturally adapted, affordable intervention for depression (Fig 8) [69]. HAP is a brief, structured intervention for moderate to severe depression, grounded in behavioral activation and incorporating psychoeducation, activity and mood monitoring, problem-solving, and social network activation [70]. HAP consists of three phases: an early phase to build rapport and introduce treatment principles, a middle phase focused on activation and problem-solving, and an ending phase addressing relapse prevention [71]. RCTs on HAP have been done in India [69,70], and Nepal [31].

#### Intervention delivery and duration.

The intervention is delivered in 6–8 weekly or biweekly sessions (30–40 minutes each) over 2–3 months [69,70,72]. Although session recommendations exist, flexibility is allowed to extend a treatment phase if the goals for that phase have not yet been achieved, delaying the transition to the next phase [71]. Sessions occur in person or by phone within primary care settings [71].

#### Helpers’ characteristics and training.

HAP is designed for delivery by non-specialist providers such as lay counsellors in India [69,70], and primary care health workers in Nepal [31]. The exact training duration for HAP is not clear, as the reviewed trials indicated the helpers receiving a combined HAP and CAP training with the duration of 10 days [31] or 3 weeks [70].

Treatment adherence was assessed by scoring a randomly selected 10% sample of audio recordings using a quality rating scale, and by supervisors reviewing self-reported treatment completion records [69].

#### Mental health conditions and populations served.

HAP has demonstrated significant reductions in depression severity and functional impairment across multiple trials. The selection criteria for the trials were depression assessed and diagnosed according to the mhGAP guidelines in Nepal [31], and moderately severe to severe depression (PHQ-9 > 14) in India [69,70].

Outcomes were measured at 3 and 12 months follow-up in Nepal [31] and India [69,70].

*Depression and Anxiety:* In India, depression was assessed with the Beck Depression Inventory (BDI-II) and the PHQ-9 [69,70]. The results showed significant reductions in depression symptoms, with remission defined as the PHQ-9 < 10 [69,70]. In India, the trials found that HAP participants maintained improvements at 12 months, with 63% achieving remission (PHQ-9 < 10) compared to 47% in the control group [69]. In Nepal, the depression significantly improved compared to the control arm and the effects were sustained at 12 months follow-up, assessed by the PHQ-9 ≥ 10 [31].

*PTSD, Substance Use Conditions, Distress:* Not evaluated.

*Functionality:* HAP also contributed to improvements in functional impairment. In India, WHODAS 2.0 assessments demonstrated better outcomes for participants receiving HAP compared to the control group 3 months follow-up, but showed only marginal effect at 12 months [69,70]. In Nepal, significant improvement in functional impairment was also observed, as measured by the WHODAS 2.0 [31].

*Other Psychological Outcomes:* The likelihood of suicidal behavior at 12 months was reduced in India among HAP participants compared to the control arm [69]. However, the difference was marginally significant; the numbers were too small to draw any conclusions as only two clients indicated suicidal attempt in both arms [69]. Finally, intimate partner violence for women and men was assessed in the studies in India; only intimate partner physical violence reported by women improved at 3 months follow-up, but no sustained effect was observed at 12 months [69,70].

### Group interpersonal therapy

#### Psychological intervention rationale and content.

Interpersonal therapy, established in the 1970s, has demonstrated efficacy in treating depression [73]. The group interpersonal therapy (IPT-G) intervention addresses grief, interpersonal disputes, role transitions, and social isolation as primary depression triggers [73]. Participants explore connections between depression and life challenges, collaboratively developing solutions with group support [73]. This intervention is not recommended for individuals with high suicide risk or those with severe mental, neurological, or substance use conditions, such as psychosis [73].

**Fig 9:**
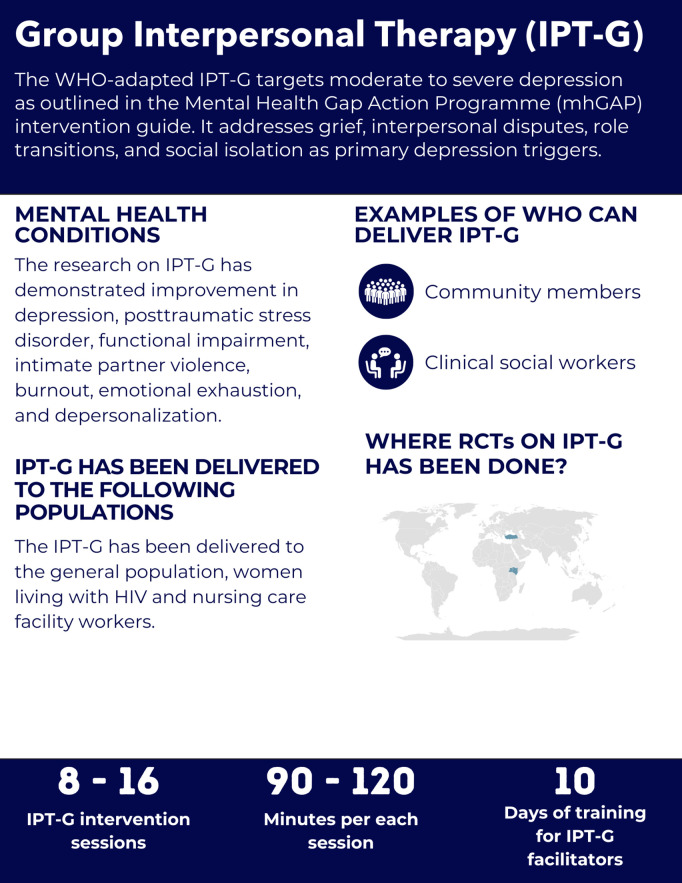
Overview of the Group Interpersonal Therapy intervention.

Although interpersonal therapy addresses various mental health conditions, the WHO-adapted IPT-G specifically targets moderate to severe depression as outlined in the mhGAP intervention guide [73]. Key RCTs on IPT-G have been done in Uganda [74,75], Kenya [76], and Turkey (Fig 9) [77].

#### Intervention delivery and duration.

The IPT-G session duration varies with group size: 90 minutes for groups of 6–10 participants and at least 120 minutes for larger groups [73]. The reported duration of the intervention in the reviewed studies ranged from 8 to 16 weekly sessions, with the duration of 60 – 90 minutes per session [74–77]. Although the sessions may differ by personal characteristics, it is essential that all members of the group, together with the facilitator, speak in the same language [73].

IPT-G follows a structured approach with four phases delivered in eight sessions, as outlined in the WHO manual; however, the evidence base includes trials with 16 sessions in Uganda and 12 sessions in Kenya [73–75]. It begins with an individual Pre-Group Phase to assess suitability and prepare participants [73]. The Initial Group Phase (session 1) introduces members, establishes a therapeutic setting, and provides psychoeducation on depression [73]. The Middle Phase (sessions 2–7) addresses interpersonal problem areas through group discussion and techniques [73]. The Termination Phase (session 8) focuses on consolidating gains and planning for future maintenance [73].

#### Helpers’ characteristics and training.

IPT-G facilitators do not require a formal mental health background but must possess strong communication and organizational skills, along with a motivation to assist others [73]. Even though the manual does not explicitly state the duration of the training, based on the reviewed RCTs, it consists of a 10-day program covering lectures, group discussions, role-playing exercises, and a final knowledge test [73,76]. Following training, facilitators conduct at least three IPT groups under supervision before transitioning to periodic supervisory support [73].

In our reviewed RCTs, individuals who delivered the IPT-G intervention were clinical social workers in Turkey [77] and community members in Uganda [74,75]. The study conducted in Kenya did not specify who the helpers were, but they were listed as nonspecialists with high school education [76].

In the reviewed studies, adherence to the intervention was assessed using checklists. While most interventions employed checklists with binary (yes/no) responses, the IPT-G treatment adherence tool utilized a 10-point Likert scale. A score of ≥ 5 was considered indicative of adherence, with supervisors verifying the ratings for accuracy [76].

#### Mental health conditions and populations served.

IPT-G has been implemented in rural Ugandan communities [74,75], among women with HIV in Kenya [76], and with nursing care facility workers in Turkey [77]. Selection criteria in Uganda were self-reported depression symptoms that met the DSM-4 criteria for MDD, study outcomes were measured at 2 week and 6 months follow-up [74,75]; MDD and PTSD diagnosis measured with MINI in Kenya, with study outcomes assessed at 3 and 6 months follow-up [76]; emotional exhaustion (score ≥ 27) and depersonalization (score ≥ 10) assessed by the Maslach Burnout Inventory (MBI) in Turkey, study outcomes measured after the intervention [77].

*Depression and Anxiety:* In Uganda (measured by the HSCL) [74,75], depression showed significant reductions sustained across 6 months follow-up, and in Kenya, assessed with the BDI-II [76].

*PTSD:* In Kenya, PTSD symptoms demonstrated significant improvement (measured via the PCL-C) [76].

*Substance Use Conditions, Distress:* Not evaluated.

*Functionality:* Functional impairment improved in Uganda (assessed using a locally developed tool) [74,75], and in Kenya, assessed by the WHODAS 2.0 at 3 and 6 months follow-up [76].

*Other Psychological Outcomes:* In Kenya, significant reductions in intimate partner violence were observed, assessed using the Conflict Tactics Scale at 3 and 6 months follow-up [76]. The study in Turkey demonstrated significant reductions in burnout, emotional exhaustion, and depersonalization, but not personal accomplishment as measured by the MBI [77].

### Common elements treatment approach

#### Psychological intervention rationale and content.

**Fig 10:**
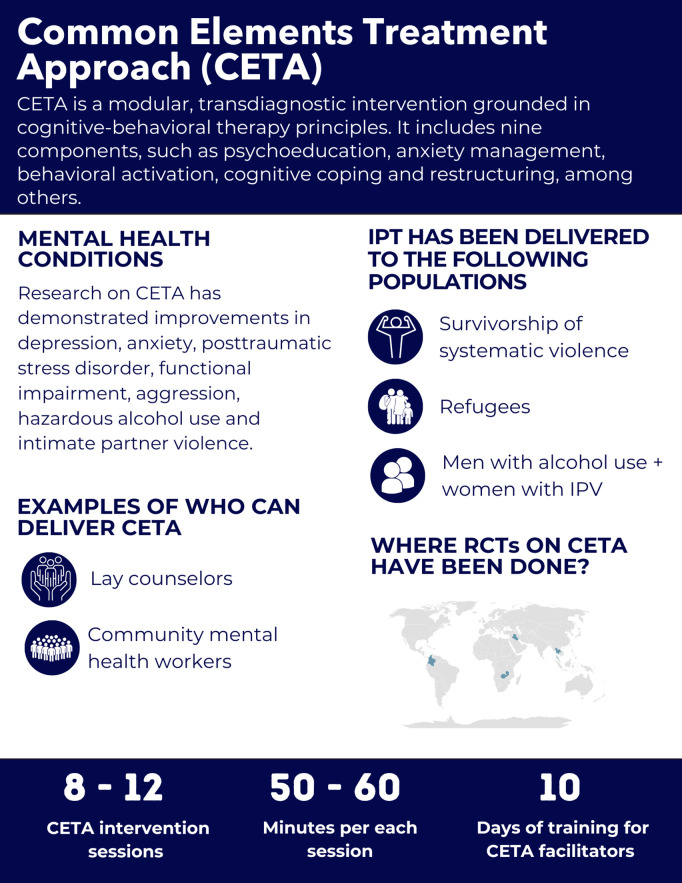
Overview of the Common Elements Treatment Approach intervention.

The Common Elements Treatment Approach (CETA) is a modular, transdiagnostic approach designed for low-resource settings based on CBT principles [78]. The standard CETA structure consists of *engagement* – encouraging participation by identifying barriers to involvement and including family members when appropriate, *psychoeducation* – introduction to CETA and normalization of problems, *anxiety management strategies*, relaxation, *behavioral activation* – focusing on pleasurable activities, *cognitive coping/ restructuring* – understanding the connection between thoughts, feelings and behaviors, *imaginal gradual exposure* – discussing challenging memories, *in-vivo exposure* – facing triggers, *suicide/ homicide/ danger assessment and planning* – assessing safety, and *screening and brief intervention for alcohol* – client encouragement to change behavior based on motivational interviewing [79]. To date, the RCTs on CETA effectiveness have been done in Thailand [80], Iraq [81], Colombia [82], and Zambia [83] (Fig 10).

#### Intervention delivery and duration.

Standard CETA consists of 8–12 sessions (each 50–60 minutes long), and a five-session brief version has been trialed in Ukraine; however, the results are pending [81,84]. Some adaptations include additional sessions on substance use and safety for violence [83].

The RCTs conducted in Thailand and Iraq provided all participants with engagement, psychoeducation, cognitive coping/ restructuring, imaginal exposure, and safety, while other components were delivered based on individual client symptoms [80,81]. The study in Colombia did not specify which components were used but allowed flexibility on modules selected by the helper, their sequence, and dosage of the intervention, although no further implementation details were provided [82]. The Zambia study did not specify whether the intervention was standardized for all participants or tailored to individual needs [83].

#### Helpers’ characteristics and training.

CETA is designed for lay counsellors without formal mental health training [85]. RCTs have been reported for implementation by lay counsellors in Thailand [80] and Zambia [83], lay psychosocial community workers in Colombia [82], and community mental health workers in Iraq [81]. Training involves a 10-day program followed by supervised sessions, progressively reducing to weekly supervision [81,86]. The apprenticeship model ensures skill development through ongoing mentorship [86].

To assess fidelity, CETA studies employed a three-tiered approach: helper self-reports and tracking sheets to document adherence to intervention strategies, review and discussion of these materials by local supervisors, and subsequent case discussions with U.S. based supervisors [80]. Other studies relied solely on supervision to ensure fidelity [82], although some used tracking logs to document treatment adherence, listing completed steps for each session along with a rating system [83].

#### Mental health conditions and populations served.

CETA has been used with Burmese refugees in Thailand [80], survivors of systematic violence in Colombia [82] and Iraq [81], and dyads of men engaged in hazardous alcohol use and women who experienced intimate partner violence (IPV) in Zambia [83]. The selection criteria were moderate to severe depressions, assessed by the HSCL-25 and posttraumatic stress, measured by the Harvard Trauma Questionnaire (HTQ) in Thailand [80]; functional impairment, depression, anxiety and trauma symptom severity (HTQ ≥ 36) in Iraq [81]; mental health symptoms, assessed by the Total Mental Health Symptoms Scale (TMHS ≥ 0.77) and functional impairment, measured by gender-specific scales in Colombia [82], and in Zambia, women had to report physical/ sexual IPV, assessed by the Severity of Violence Against Women Scale (SVAWS, physical/ sexual violence subscale ≥ 38); and men self-reported hazardous alcohol use, measured by AUDIT ≥ 8 [83].

Outcomes were measured at 4 months in Iraq [81] and 12 months follow-up in Zambia [83]. The follow-up period in the studies done in Thailand [80] and Colombia [82] was not clearly defined, but follow-up likely referred to the post-intervention assessment.

*Depression and Anxiety:* The intervention has significantly reduced depression and anxiety in the 3 out 3 studies in which they were evaluated: Thailand [80], Colombia (only in one out of two study sites) [82], and Iraq [81], as measured by the HSCL-25 and TMHS in Colombia.

*PTSD:* Improvement in post-traumatic stress symptoms was observed in all three studies where it was evaluated; in Thailand [80], assessed by the HTQ, and in Colombia [82], measured by the HTQ and the PCL-C, the only significant outcome found across two municipalities. In Iraq, trauma-related symptoms were also assessed using the HTQ, showing significant improvement [81].

*General Psychological Distress:* Not evaluated.

*Substance Use Conditions:* Adaptations in Zambia included additional focus on substance use, and showed associations with reductions in hazardous alcohol use in men, assessed by the AUDIT ≥ 8 at a 12 months follow-up [83]. The CETA had no significant improvement between intervention and control arms in alcohol use among problem drinkers in Thailand, measured by AUDIT [80].

*Functionality:* Functional impairment improvement was observed in Iraq [81], Colombia (only in one out of two study sites) [82], and Thailand [80], as measured by the locally developed functioning scales.

*Other Psychological Outcomes:* In Zambia, CETA was associated with reductions in intimate partner violence, measured by the SVAWS [83]. Additionally, in Thailand [80], aggression, measured by the Aggression Questionnaire, was also significantly reduced.

### Cognitive processing therapy

#### Psychological intervention rationale and content.

**Fig 11:**
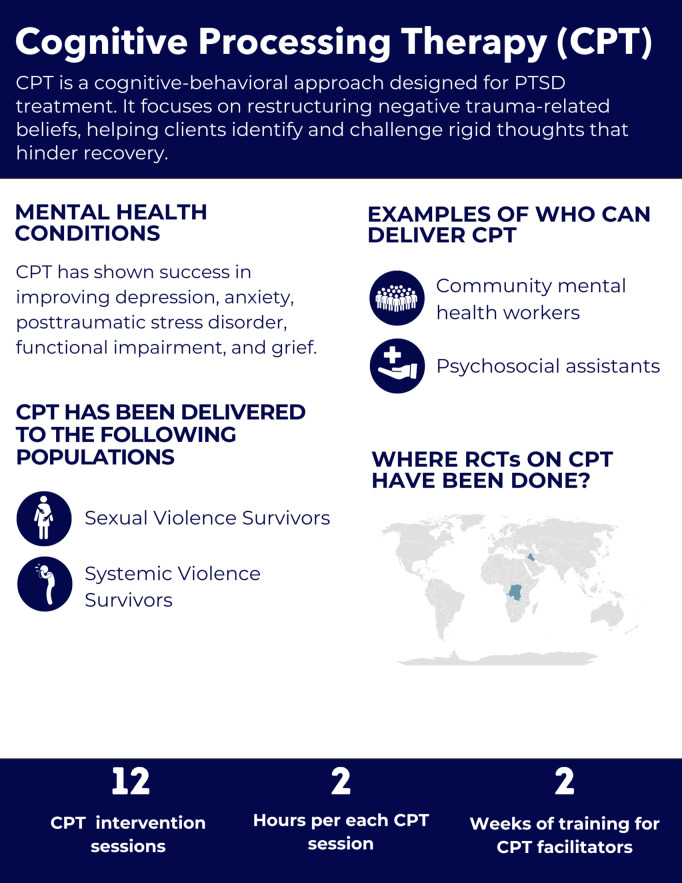
Overview of the Cognitive Processing Therapy intervention.

Cognitive Processing Therapy (CPT), is a cognitive-behavioral approach designed for PTSD treatment [87]. It focuses on restructuring negative trauma-related beliefs, helping clients identify and challenge rigid thoughts that hinder recovery [88]. The RCTs up to date have been done in the Democratic Republic of the Congo [89], and Iraq (Fig 11) [81,88].

#### Intervention delivery and duration.

CPT is structured around linking traumatic events to cognitive distortions and emotional responses [88]. The therapy involves identifying and modifying inaccurate trauma-related beliefs across key domains: safety, trust, power, control, esteem, and intimacy [88]. It can be delivered individually or in groups, in 12 two-hour sessions [81,88,90].

#### Helpers’ characteristics and training.

CPT training includes a two-week program provided by U.S.-based practitioners or free online courses. The training follows an apprenticeship model, with local and international supervision ensuring fidelity [81,88,90]. In the reviewed RCTs, CPT was implemented by community health workers (including medics and nurses) in Northern Iraq [81], community mental health workers in Northern Iraq [88] and psychosocial assistants in Congo [89]. The helper training duration was described 2 weeks [88,89].

In the reviewed studies, fidelity was assessed using supervisor-completed checklists and ratings of knowledge and skills [89], as well as through helper self-reports, supervisor notes, and reviews conducted by the intervention trainer [81].

#### Mental health conditions and populations served.

The effectiveness of CPT has been evaluated in diverse populations affected by trauma, including survivors of systematic violence in Iraq [81,88], and female survivors of sexual violence in Congo [89].

The inclusion criteria were functional impairment, depression, anxiety and trauma symptom severity (HTQ ≥ 36) in Southern Iraq [81], witnessing or experiencing sexual violence, mental health symptoms (combination of HSCL-25 and HTQ items ≥ 55) and functional impairment (assessment of the difficulty of doing different tasks, score ≥ 10) in Congo [89], depression (HSCL-25 including 15 standard and 5 local symptoms, score ≥ 20) in Northern Iraq [88].

Outcome time points were approximately 4 months in Southern Iraq [81], 1 and 6 months after the treatment was completed in Congo [89], and on average, 5.5 months follow-up in Northern Iraq [88].

*Depression and Anxiety:* In Southern Iraq, CPT resulted in significant improvements in depression symptoms, as measured by the HSCL-25 [81], moderate effects were observed in the Northern part of Iraq when compared to the CPT controls [88]. In contrast, the study conducted in the Democratic Republic of the Congo found substantial reductions in combined depression and anxiety symptoms, assessed by the HSCL-25, which were maintained at 6 months follow-up, highlighting the sustained benefits of the intervention [89]. Significant effects on anxiety were also found in Northern Iraq [88]. In Southern Iraq, there were small to no effects found on anxiety, assessed by HSCL-25 [81].

*PTSD:* For PTSD, participants in the Democratic Republic of the Congo showed significant improvements, as assessed using the HTQ [89]. In Northern Iraq, moderate effects were observed when compared to the CPT controls [88]. The effects of trauma were also observed in Southern Iraq, assessed by the HTQ [81].

*Substance Use Conditions, General Psychological Distress:* Not evaluated.

*Functionality:* CPT demonstrated efficacy in improving functional impairment in Northern Iraq [88] and Congo [89], but no significant improvement was found in Southern Iraq [81], as measured using locally developed scales.

*Other Psychological Outcomes:* In Northern Iraq, the Inventory of Traumatic Grief was used to assess traumatic grief; CPT demonstrated moderate effects for grief when compared to the intervention controls [88].

### Thinking healthy programme

#### Rationale and content.

**Fig 12:**
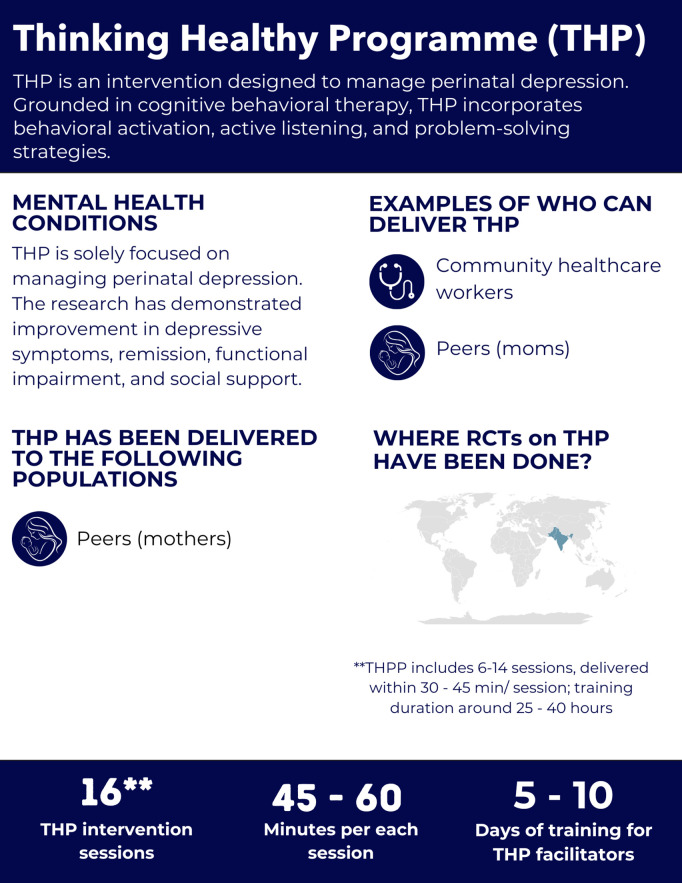
Overview of the Thinking Healthy Programme intervention.

The Thinking Healthy Programme (THP) is an intervention designed to manage perinatal depression (Fig 12) [91]. Grounded in cognitive behavioral therapy, THP incorporates behavioral activation, active listening, and problem-solving strategies [91]. Sessions are structured around key perinatal stages: preparing for the baby (weekly, 14–40 weeks pre-birth), baby’s arrival (biweekly, 3rd–5th week post-birth), early infancy (monthly, 2nd–4th month post-birth), middle infancy (monthly, 5th–7th month post-birth), and late infancy (monthly, 8th–10th month post-birth) [91]. RCTs were conducted in Pakistan [92,93] and India [94].

#### Intervention delivery and duration.

THP consists of five modules delivered across 16 sessions, each lasting 45–60 minutes [91]. Though implemented in primary care settings, adaptations for peer delivery—referred to as THPP—have also been tested in Pakistan and India [93,94]. The THPP delivery can be completed in 6–14 sessions, each lasting 30–45 minutes [93].

#### Helpers’ characteristics and training.

THP can be delivered by individuals without formal mental health training with ongoing supervision [91]. Training lasts at least five days, with a ten-day program considered ideal [91]. The THPP training duration was reported to be 25–40 hours long [94]. Trainers and supervisors should have at least 12 months of THP experience and undergo monthly supervisory sessions [91]. The reviewed RCTs implemented THP through lady (community) health workers in Pakistan [92], and peer mothers in India and Pakistan [93,94]. In Indian and Pakistan trials, peers were mothers with strong communication skills and similar sociodemographic backgrounds [93,94]. In India, peers were referred to as *Sakhi*, meaning ‘friend’ in Hindi, and in Pakistan, *Razakaars,* which means ‘volunteer helpers.’ [93,94].

THP treatment adherence was assessed through supervision sessions and competency evaluations [93]. Another study tracked session frequency and duration, as well as the number of participants who completed or failed to complete the sessions [94].

#### Mental health conditions and populations served.

Inclusion criteria were major depressive episode based on DSM-4 criteria in Pakistan [92], depression (PHQ-9 ≥ 10) in India and Pakistan [93,94].

Outcome time points were 6 and 12 months in Pakistan [92], 3 and 6 months in India and Pakistan [93,94].

*Depression and Anxiety:* THP has demonstrated significant benefits across multiple settings in reducing depressive symptoms and achieving remission. In the THP study in Pakistan, depression was measured using the Hamilton Depression Rating Scale (HDRS), showing a significant reduction in depressive symptoms at 6 and 12 months follow-up [92]. In the THPP trials conducted in Pakistan and India, remission was assessed using the PHQ-9, with remission defined as PHQ-9 < 5 [93,94]. At three months follow-up, the intervention group experienced lower PHQ-9 score compared to the control group; however, the benefits were not significant at 6 months follow-up in Pakistan; in India, remission rates were 73% versus 60%, demonstrating moderate effects [93,94].

*PTSD, Substance Use Conditions, General Psychological Distress:* Not evaluated.

*Functioning:* The THPP study in Pakistan and India did not show improvements in disability and functioning, assessed by the WHODAS 2.0 at 6 months follow-up [93,94]. However, disability and functionality improved in Pakistan, assessed by the Brief Disability Questionnaire and Global Assessment of Functioning Scale at 6 and 12 months follow-up [92].

*Other Psychological Outcomes:* Social support benefits were reported in both THPP and THP studies, evaluated using the MSPSS [92,94]. The THPP in Pakistan showed improvements in social support at 6 months but not at 3 months follow-up, whereas THPP in India improved social support at both 3 and 6 months follow-up [93,94]. The THP study in Pakistan demonstrated significant benefits for social support at 6 and 12 months follow-up [92].

## Discussion

This review aims to support global mental health practitioners, researchers, and policymakers in selecting psychological interventions for low-resource settings. We reviewed 10 interventions and PFA, summarizing their rationale, content, target conditions, populations, processes, duration, and helper training.

### Intervention rationale and content

Despite being delivered across diverse settings and by various types of providers, the reviewed interventions share several overlapping therapeutic elements, as demonstrated by other literature reviews [95]. Many incorporate behavioral activation strategies, as seen in HAP, and other interventions, such as PM+ and the Friendship Bench, emphasize problem-solving techniques. SH + is distinct in its use of an Acceptance and Commitment Therapy. Some interventions integrate specialized components, such as motivational interviewing for substance use, featured in both CAP and CETA. Exposure-based strategies, used for trauma-related conditions, are present in CPT and CETA. Notably, CETA stands out as a modular intervention, offering a flexible structure with clearly defined components tailored to a range of mental health conditions.

### Intervention delivery and duration

The length and mode of delivery of psychological interventions can significantly influence their feasibility, particularly in humanitarian settings where resources are often constrained. PM+ and SbS are both structured to be delivered in five sessions (90 min vs. 30 min per session, respectively) [38,57–60]. However, it is important to note that PM + can be delivered in person, whereas SbS requires a digital platform and is the only reviewed intervention that is self-directed.

Some interventions, like PM + , offer flexibility by being delivered in person, in groups [38,39], or remotely [96]. The HAP can be administered face-to-face or by phone, allowing for adaptation in different settings [71]. Increasingly, interventions are shifting towards digital platforms. SbS is a web-based, self-paced program designed for smartphone use, expanding accessibility to populations that may lack in-person services [57–60]. However, the use of online interventions must consider factors such as internet reliability and digital literacy, particularly in low-resource settings where access to technology may be limited [58].

A critical question when selecting an intervention is determining the minimum effective dose needed, particularly in humanitarian settings. Studies on PM+ have reported an average attendance of 2.86 sessions in Pakistan [41] and 3.96 in Jordan [51], yet both have demonstrated positive mental health outcomes. Understanding how many sessions are necessary for meaningful improvement could help refine intervention models to maximize impact while minimizing the burden on clients and providers. Additional research should also explore whether booster sessions could help sustain benefits over time. Answering these questions is crucial for refining interventions, optimizing resource allocation, and ensuring sustainable mental health support in low-resource settings.

One common trend observed was high dropout rates, particularly in digital interventions like SbS, which have reported attrition rates of 59%–76% [57–60]. Although SbS was designed for internet-based delivery, technological limitations in Lebanon [58], and low engagement in China [59] contributed to substantial participant dropout. High attrition has also been observed in non-digital interventions. For instance, the CETA trial in Colombia experienced elevated dropout rates, likely influenced by ongoing violence and insecurity in the region [82]. In Thailand, attrition was higher in the control arm, potentially due to population mobility [80]. In contrast, IPT-G trials showed relatively balanced sex distributions and lower attrition rates; for example, no participant loss was reported in Turkey [77].

Such levels of attrition can bias outcomes and limit generalizability. Future research should prioritize testing interventions in more diverse populations and real-world contexts. Moreover, implementation efforts must enhance user engagement, ensure reliable internet connectivity, and address barriers related to digital literacy.

### Helpers’ characteristics and training

Selecting suitable providers is critical for the success of psychological interventions, particularly in low-resource settings where trained professionals are scarce. Key facilitators of successful implementation include providers’ competencies, a positive attitude, beliefs that mental health conditions can be effectively treated, and a strong relationship with the community [97]. Recruiting providers with sociodemographic characteristics similar to those of the target population can enhance accessibility and comfort, making mental health services feel more approachable [98]. However, over-familiarity within the community may raise concerns about confidentiality and stigma, requiring a balance between accessibility and privacy [98].

Client characteristics also affect intervention success. Barriers such as poverty, unemployment, unstable housing, and low literacy can hinder participation [97]. These challenges may impact attendance, comprehension, and accessibility. Some interventions, such as FB and THP, address these issues by training helpers from within the community, which can enhance engagement and acceptability [97]. Furthermore, most studies recruit participants based on the predetermined cutoff scores for specific conditions (e.g., PHQ-9 for depression, GAD-7 for anxiety, etc.) and intervention is delivered as a standardized package with limited or no customization. This limits the ability to tailor strategies, for example, using behavioral activation for individuals experiencing social isolation or problem-solving techniques for those facing significant life stressors.

Training and supervision are essential for ensuring fidelity of interventions. Studies highlight the importance of supervision in improving skills, enhancing credibility within the target population, and preparing individuals for potential barriers when delivering the intervention [98]. Adherence to the intervention approaches in the reviewed studies was primarily assessed using intervention checklists/ forms completed by supervisors [33,36,37,50,51,53,89], independent observers [49], through self-report [34,35] or through competency assessments [50]. Other studies included a combined approach—using self-reported measures together with supervisor review [80,81]. Some or all sessions were audio recorded to facilitate the scoring the checklists more easily [30,66,69,94]. Standardizing fidelity procedures across interventions is critical to ensure that programs are delivered as intended and that their effectiveness can be compared across different trials, as variations in fidelity may have an impact on the measured outcomes. To improve adherence and quality assurance, fidelity evaluations should be integrated with competency assessments, enabling evaluation not only of intervention protocol adherence but also of the helper mental health support skills required for effective delivery. Recently, the WHO incorporated the Ensuring Quality in Psychosocial and Mental Health Care (EQUIP) competency assessment tools into the latest SH+ manual as a standardized mental health provision quality standard [99]. These tools provide a structured and standardized approach to evaluating the competencies necessary to deliver SH + , using role-plays to assess key skills and identify specific behaviors that require improvement [100]. The competency assessment tools are adaptable for use with any mental health intervention, providing a mechanism to ensure the quality of care and safeguard against potential harm to the intervention recipients [99,101–103].

### Mental health conditions and populations served

**Fig 13:**
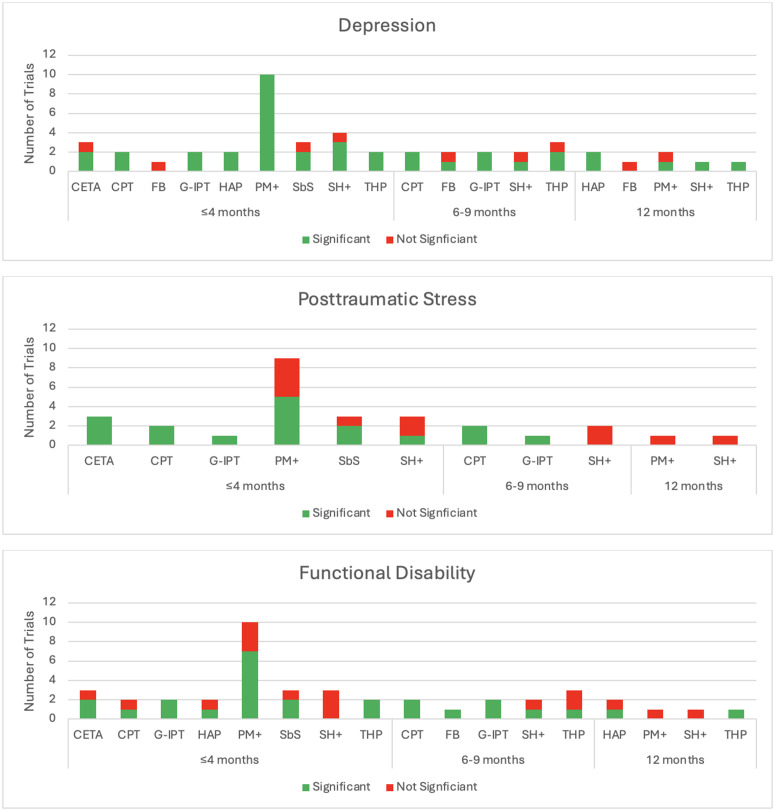
Number of trials with significant or non-significant outcomes by intervention and period of final assessment for depression, posttraumatic stress and functional impairment. *This figure includes data collected across multiple time points, which may appear in more than one section of the graph. **Statistical significance is denoted by *p* < 0.05, *p* < 0.01, and *p* < 0.001. ***Functional impairment reflects participants' overall level of functioning and disability. Abbreviations: CETA, Common Elements Treatment Approach; CPT, Cognitive Processing Therapy; FB, Friendship Bench; G-IPT, Group Interpersonal Therapy; HAP, Healthy Activity Program; PM+, Problem Management Plus; SbS, Step-by-Step; SHt, Self Help Plus; THP, Thinking Healthy Programme.

Although all the reviewed interventions have demonstrated success in improving mental health outcomes, the choice of intervention needs to be carefully considered, taking into account the target population’s mental health needs as indicated in the WHO’s psychological intervention implementation manual [13]. Most of the data on benefits of these interventions is limited to short time periods such within 4 months, but few studies have information for sustained benefit at one year or longer (Fig 13). The same intervention effective in a particular setting or with a specific population may not be successful in a different context. For example, SH + has been successful in humanitarian settings, improving psychological distress, PTSD, depression, and self-identified problems [33–37]. However, an RCT in Italy with care home workers found no effect, highlighting the importance of contextual adaptation [104].

Regardless of the approach selected, suicide risk assessment is critical. While these low-intensity interventions are not designed to manage high suicide risk, most include self-harm screening. PM+ integrates suicide risk assessment in every session, guiding helpers on assessing ideation, plan, and intent [38]. Similarly, CAP, CETA, FB, HAP, IPT-G, and SH+ provide structured guidance on suicide discussions and risk assessment, relying on referrals for clients requiring more intensive care [29,65,73,78,105]. Most of these interventions rely on the assumption that referrals should be given to clients with significant psychological challenges for longer-term care [106]. However, PFA, CPT, and THP lack suicide risk assessment protocols, highlighting a gap in structured identification and support for at-risk individuals. Establishing standardized suicide risk assessment across all interventions would improve early identification and referral, ensuring client safety in diverse implementation settings.

All reviewed interventions demonstrated benefits for reducing depression, except for CAP, which did not target depression as an outcome. An important consideration across these trials is the variability in how depression was assessed. While most studies used the PHQ-9, the cut-off scores to define depression varied substantially. For example, the HAP is not recommended for individuals with mild depression (PHQ-9 score 10–14), as symptoms in this range may remit without intervention [71]. Such variability in the assessments highlights the need for greater consistency in how depression is defined and measured across trials.

Significant improvements in anxiety symptoms were observed across all intervention final assessments, except for HAP, SH + , IPT-G, THP, and CAP; however, it is important to note that anxiety was not assessed in all the trials, except for SH + . CETA also showed positive effects in reducing substance use. Improvements in functioning were reported for most interventions, except for CAP—again, consistent with its primary focus on alcohol-related outcomes rather than functional impairment, which is a significant limitation requiring additional research. Several interventions, including SH + , PM + , SbS, IPT-G, CETA, and CPT, demonstrated effectiveness in addressing trauma-related symptoms. Overall, while interventions varied in their targeted outcomes, most showed robust effects across common mental health domains, particularly depression, anxiety, and trauma.

CETA and CAP showed improvement in the alcohol use conditions in Zambia [83], and India, but not in Nepal. As a potential explanation, the authors indicated that participants with both harmful (type of drinking that has caused physical, social and mental harm) [29], and dependent drinking (combination of harmful behaviors and thoughts together with physical harm, resulting in daily alcohol consumption) [29] were included in the Nepal study, unlike in India where only harmful drinking was included, which could have had an impact on the intervention effectiveness [31]. Furthermore, the study in Nepal did not reach its target sample size, and the helpers were all females, which could have had an effect on client engagement in the intervention, as all the clients were men [31]. Drawing on similar studies from Nepal, the authors suggested that integrating evidence-based mental health support into primary care may be sufficient for some individuals, leaving little added benefit from additional services [31].

Contextual factors were found to be essential to ensure intervention success. For example, CETA showed greater improvements than CPT in southern Iraq, potentially due to contextual differences: CPT control participants were in relatively safer, urban areas with better access to mental health services, possibly reducing the contrast between study arms [81]. In Colombia, more substantial CETA effects were found in Buenaventura compared to Quibdó, despite being similar settings; potentially due to greater unmet basic needs in Quibdó, distinct patterns of violence (guerrilla vs. drug trafficking), and more limited geographic access [82]. Overall, these findings underscore the importance of contextual, structural, and methodological factors in interpreting intervention effectiveness across diverse settings.

From a population’s perspective, it is unclear whether these interventions are equally effective for men and women. Most RCTs had predominantly female participants, such as the 82% female sample in the PM+ study in Nepal [50], and similar proportions in FB trials in Zimbabwe [66,67], the SH + RCT in China [35], and the HAP trial in Nepal [31]. Some studies exclusively focused on women, such as SH+ trial in Uganda [33], PM+ trials in Kenya [43] and Pakistan [49].

The CAP trials were the exception in terms of participant demographics, with outcomes based predominantly on male samples—83% of participants in Nepal were men [31], and all participants in India were men [28,30]. Although CAP is not designed as a sex-specific intervention, further research is needed to assess whether its effectiveness is comparable for women. Additionally, SH+ trials in Western Europe primarily included men, with 72% of participants identifying as male [36,37]. Such patterns raise important questions about the generalizability of findings, particularly for male populations. Further research is needed to explore sex differences in intervention effectiveness and to ensure that mental health services are appropriately tailored to meet the needs of both men and women.

Finally, comprehensive care approaches should also account for populations beyond the primary focus. For instance, THP targets perinatal depression, yet men and male partners also experience psychological distress during their partner’s pregnancy [107]. If available, addressing their needs through resource allocation or referral mechanisms could enhance the intervention’s impact. Conducting community resource mapping and coordinating with local organizations can facilitate this process.

At the same time, the generalizability of these interventions may be constrained by the specific characteristics of the populations studied. For example, despite strong effects on perinatal depression, generalizability may be limited. Nearly all participants in the Pakistan (100%) and India (99%) trials were married [93,94], a factor known to influence depression risk due to established links between partner support and maternal well-being [108]. Additionally, most participants had primary or secondary education (Pakistan: 66%, India: 70%) [93,94], which may impact intervention engagement and health-seeking behaviors. Furthermore, all THP trials have been conducted in South Asian contexts, and more evidence is needed from other settings to assess the effectiveness of the intervention across diverse populations.

### Cultural adaptation

Most manuals and trials mentioned translation, visual adaptation, and alignment with local conceptualizations of distress. However, the adaptation processes varied in scope and depth, with some interventions undergoing extensive modifications while others applied only minimal changes. Two systematic reviews reported that culturally adapted interventions were generally more effective than non-adapted ones, improving acceptability, effectiveness, and client satisfaction [109,110]. FB was developed around Shona idioms of common mental health conditions, making it inherently culturally embedded [65]. However, even though adaptation is widely acknowledged as beneficial, there remains limited guidance on standardized processes. Frameworks such as the Mental Health Cultural Adaptation and Contextualization for Implementation (mhCACI) provide structured methodologies, that emphasize core mechanisms of action, including training, supervision, and fidelity assessments [111]. mhCACI, used in adapting PM+ in Nepal, outlines key adaptation steps, including translation, expert review, community engagement, and iterative revisions based on implementation feedback [111]. The WHO Psychological Interventions Implementation Manual outlines essential and optional steps for adaptation, depending on available resources, such as literature review, quality assessment, translation of the intervention materials, expert review, evaluation and reaching an agreement on proposed adaptations, re-evaluation of the content during training, and piloting the adapted version [13].

Cultural adaptation efforts often include translating materials and integrating culturally relevant visual aids. For instance, the PM+ and SH+ intervention manuals have been translated and adapted into multiple languages, incorporating pictorial elements to ensure therapeutic relevance across diverse cultural contexts. Despite these efforts, some implementers claim that the cultural adaptation in the field is limited, lacking specific guidance on how to adapt an intervention [112]. Contextual adaptations are also necessary. For example, THP focuses on perinatal depression but does not account for miscarriage, neonatal death, or stillbirth experiences. Given that women who experience miscarriage may develop a mental health condition [113], contextualizing THP for common adverse reproductive outcomes in a specific setting and incorporating culturally relevant grief practices may improve the local fit of the intervention.

Another challenge is the geographic limitation of adapted interventions, which affects scalability. Interventions like PM + , IPT, SbS, THP, and CETA, have been tested across multiple countries; however, others, such as HAP, CAP, and FB have only undergone trials in one or two locations. Some interventions, such as CPT, have been adapted from HICs for use in LMICs. Alternatively, interventions originally developed in LMICs—such as FB, CETA, and PM + —are now being transferred and adapted for implementation in HICs. Notable examples include Friendship Bench integration into New York community health centers and Washington, D.C. community centers, CETA’s use in telephone-based support for adults living with HIV in Alabama, and PM+ implementation within community members living in New York and a few European countries delivered remotely [55,114–116]. This exchange of interventions between LMICs and HICs, referred to as *reciprocal innovation*, has the potential to enhance equitable mental health access globally [117]. Addressing these gaps requires prioritizing cultural adaptation in implementation research, ensuring that interventions remain contextually relevant while retaining their evidence-based components.

### Integration in existing programs

Integrating psychological interventions into existing programs is crucial for their sustainability and effectiveness. Evidence suggests that integrating mental health services into broader health and social systems can improve outcomes [118]. For example, SH + has been successfully integrated into humanitarian programs in Uganda by leveraging partnerships in the health, protection, and livelihood sectors to expand its reach [119]. In Europe, SH + has also been embedded within a stepped care model. Two RCTs in Spain and Italy evaluated this approach, using SH+ as the initial intervention, followed by PM+ for those with persistent psychological distress [120,121]. In both studies, participants in the intervention arm received SH+ along with an illustrated guide, *Doing What Matters in Times of Stress* [32]. The Italian study additionally included PFA before offering PM+ to individuals still experiencing distress [121]. The stepped care model showed significant reductions in anxiety and depression, measured by the PHQ-Anxiety and Depression Scale (PHQ-ADS), as well as improvements in PTSD symptoms (PCL-5) and functioning and well-being (PSYCHLOPS) [120,121]. Notably, in Italy, these effects were sustained 21 weeks post-intervention, highlighting the model’s long-term benefits [121].

The successful integration of psychological interventions requires strong collaboration among local communities, governments, and service providers. Poor coordination and communication among stakeholders can limit support, learning opportunities, and resource-sharing [122]. A qualitative study on PM+ in Jordan highlighted service provision gaps and communication barriers as key obstacles to scalability [123]. Effective scale-up depends on centralized tracking systems to coordinate efforts and prevent service duplication [123]. While integration enhances sustainability, it demands strategic planning, strong partnerships, and local adaptation to maximize long-term impact.

## Strengths and limitations

This review synthesizes information on 10 psychological interventions and PFA, integrating findings from intervention manuals and RCTs conducted in low-resource settings. To our knowledge, this is the first attempt to create a comprehensive overview and infographics to assist in intervention selection.

However, there are limitations. First, we focused only on RCTs in LMICs and low-resource settings in HICs, excluding other research that could provide additional insights. Second, our intervention selection was based on expert recommendations rather than a systematic inclusion process. Third, while we refer to these as “interventions,” PFA and CETA may be better categorized as approaches. Fourth, RCT findings may not always translate to real-world settings, as implementation studies were not reviewed. Fifth, while some studies reported cutoff scores for the assessment tools used, other RCTs did not. The absence of this information may limit the clinical relevance and real-world applicability of the findings.

## Conclusion

This paper guides policymakers, program designers, and implementers in selecting psychological interventions suitable for low-resource settings. Understanding intervention requirements helps funders and governments allocate resources efficiently, supporting large-scale implementation. With numerous interventions available, choosing the right one can be a complex process. This review is a roadmap outlining interventions based on format, duration, and target conditions.

We recommend developing a centralized, frequently updated database of psychological interventions in LMICs, listing new research, target populations, and implementation strategies. Some organizations have initiated such efforts, but scaling global mental health requires expanding these repositories to maximize the use of existing, adapted, and tested interventions.
